# Loss of CREST leads to neuroinflammatory responses and ALS-like motor defects in mice

**DOI:** 10.1186/s40035-019-0152-1

**Published:** 2019-04-02

**Authors:** Cheng Cheng, Kan Yang, Xinwei Wu, Yuefang Zhang, Shifang Shan, Aaron Gitler, Anirvan Ghosh, Zilong Qiu

**Affiliations:** 1 Institute of Neuroscience, State Key Laboratory of Neuroscience, CAS Center for Excellence in Brain Science and Intelligence Technology, University of Chinese Academy of Sciences, Chinese Academy of Sciences, Shanghai, 200031 China; 2 Xiangya Hospital, Central South University, Changsha, 410008 China; 3 Department of Genetics, Stanford University School of Medicine, Stanford, CA 94305 USA; 4 Research and Early Development, Biogen, Cambridge, MA 02142 USA

**Keywords:** ALS, CREST, Neuroinflammation, Cytokine, Microglia

## Abstract

**Background:**

Amyotrophic lateral sclerosis (ALS) is a late onset neurodegenerative disease with fast progression. ALS has heavy genetic components in which a series of genetic mutations have been identified. In 2013, Mutations of the *CREST* gene (also known as *SS18L1*), which functions as a calcium-regulated transcriptional activator, were found in sporadic ALS patients. However, the pathogenic causality and mechanisms of ALS-associated mutations of *CREST* remain to be determined.

**Methods:**

In this study, we constructed CREST knockout and Q394X knock-in mice with CRISPR/Cas9 system. Using biochemical and imaging tools, we illustrated core pathological phenotypes in CREST mutant mice and claimed the possible pathogenic mechanisms. Furthermore, we also observed locomotion defects in CREST mutant mice with behavioural tests.

**Results:**

We demonstrate that ALS-related CREST-Q388X mutation exhibits *loss-of-function* effects. Importantly, the microglial activation was prevalent in CREST haploinsufficiency mice and Q394X mice mimicking the human *CREST* Q388X mutation. Furthermore, we showed that both CREST haploinsufficiency and Q394X mice displayed deficits in motor coordination. Finally, we identified the critical role of CREST-BRG1 complex in repressing the expression of immune-related cytokines including *Ccl2* and *Cxcl10* in neurons, via histone deacetylation, providing the molecular mechanisms underlying inflammatory responses within mice lack of CREST.

**Conclusion:**

Our findings indicate that elevated inflammatory responses in a subset of ALS may be caused by neuron-derived factors, suggesting potential therapeutic methods through inflammation pathways.

**Electronic supplementary material:**

The online version of this article (10.1186/s40035-019-0152-1) contains supplementary material, which is available to authorized users.

## In brief

Cheng et al. discover that neuronal loss of CREST reduces the protein level of FUS, de-represses transcriptional inhibition of chemokine genes which in turn causes microglial activation and proinflammation, and ultimately leads to axonal degeneration of motor neurons and impairment of locomotion.

## Background

Amyotrophic lateral sclerosis (ALS) is one of the most severe neurodegenerative diseases characterized by the fast progressive degeneration of motor neurons in the central nervous system (CNS). Usually within 3–5 years of disease onset, patients suffer multiple symptoms including muscle atrophy, paralysis and respiratory failure, during which there are limited therapeutic approaches to alleviate disease symptoms. Moreover, clinical features of ALS appeared great heterogeneity, meaning that ALS patients may experience motor neuron death onset in different regions of CNS, such as the spinal cord, brainstem or motor cortex [1]. ALS has heavy genetic components in which a series of genetic mutations have been identified. Since ALS-causing mutations in *SOD1* gene were reported in 1993 [2], more than 50 ALS-associated genes have been subsequently reported, including *TARDBP*, encoding TAR DNA-binding protein 43 (TDP43) [3], *FUS* [4, 5], *C9orf72* [6, 7]. With the development of next-generation sequencing (NGS) technology, many previously unknown ALS-linked mutations have been certified via whole-exome sequencing. Several de novo and inherited mutations in *CREST* have been recently reported in ALS patients via NGS-based whole-exome sequencing or target gene sequencing approaches [8–10], suggesting that *CREST* may be a potential ALS-causing gene. Among all the mutations, we focus on one de novo missense mutation, CREST-Q388X, which leads to a truncation that lacks nine amino acids in the C-terminus [8].

*CREST* is characterized as a calcium-regulated transcriptional activator of which C-terminus is responsible for gene activation through interacting to CREB-binding protein (CBP) [11]. Whereas the N-terminus of CREST protein has auto-inhibitory function via interacting with the chromatin remodeling BRG1 complex which in turn recruits histone deacetylase complex HDAC1 to inhibit gene transcription [12]. Although previous study showed that both loss of CREST and overexpression of Q394X (the corresponding truncation of mouse *Crest* homolog) or I123M mutant blocked the depolarization-induced dendritic outgrowth in cultured neurons [8, 11], whether mutations of *CREST* lead to ALS-like phenotypes in vivo remain to be determined.

Previous publications have demonstrated that non-neuron-involved chronic inflammatory responses play a critical role in the pathogenesis of ALS [13–16]. During neuroinflammatory responses, microglial activation, astrogliosis and infiltration of peripheral immune cells are the main observable pathophysiological hallmarks [17–24], and subsequent production of neurotoxic factors such as tumor necrosis factor α (TNF-α) and interleukin 1β (IL-1β) can deteriorate disease progression [25, 26]. As resident macrophages in CNS, microglia perform the first defense line of innate immune system, and thereby the excess activation of microglia predominantly conducts sustained neuroinflammatory responses that contribute to the progression of ALS or other neurodegenerative diseases [27–29]. Taken together, we hypothesize that ALS-linked CREST mutations may contribute to the aberrant neuroinflammation in vivo which in turn causes ALS-like phenotypes in mice.

In this study, we show the impaired protein stability of Q388X mutant compared to wild-type (WT) CREST in contrast to previous report [30]. Importantly, we demonstrate that microglial appearance exhibits activated morphology including the enlargement of cell bodies and the decreased complexity of processes in both CREST knockout (KO) mice and homozygous *Crest* ^*Q394X/Q394X*^ (Abbreviated as Q394X) mice, suggesting that CREST haploinsufficiency or ALS-related mutation leads to microglial activation and sustains the proinflammatory state in CNS. In agreement with ALS-like phenotypes, we also show significant denervation of tibialis anterior (TA) muscles in CREST KO mice and locomotion impairment in both CREST KO and Q394X mice. Finally, considering the specific expression of CREST in neurons, we suggest that upregulation of two important chemokines, *Cxcl10* and *Ccl2*, in neurons lack of CREST may contribute to inflammatory activation in CNS. Taken together, we demonstrate that neuronal loss of CREST function caused by ALS-linked mutation induces the alteration of immune-related genes expression which leads to microglial activation and sustained proinflammatory responses, which in turn impair motor neurons and motor behaviors of mutant mice.

## Methods

### Plasmid construction

The human *CREST* coding sequence was synthesized and then subcloned to FUGW vector (Addgene Catalog #14883) with HA tag by enzyme digestion approach. We constructed the HA-Q388X mutant-expressing plasmid from HA-CREST WT-expressing plasmid with the KOD-Plus-Mutagenesis Kit (Toyobo) according to manufacturer’s instructions. The harboring vector of shRNA targeting mouse CREST or BRG1 is pFUGW-H1 empty vector (Addgene Catalog #25870). All the FUGW and pFUGW-H1 plasmids were packaged into lentivirus for high transfection efficiency in cultured primary neurons.

### Animals and ethics statement

The C57BL/6 *Mus musculus* was the experimental model in this study. All mice were maintained in a specific pathogen free (SPF) unit under constant temperature, humidity, ventilation and automatic light cycles. All genotypes, including CREST knockout and Q394X knock-in, used in experiments were confirmed by Sanger sequencing. CREST knockout and Q394X knock-in mice were constructed with CRISPR/Cas9 system on the genetic background of C57BL/6 mouse. The sequences of small guide RNA (sgRNA) for CREST knockout or Q394X knock-in mice and the repair donor for Q394X knock-in mice are shown in the Key Resources Table (Additional file 1: Table S1). All animal-involved experiments were approved by the Biomedical Research Ethics Committee at the Shanghai Institutes for Biological Science (CAS). The use and care of animals were in accordance with the guidelines of this committee.

### Primary cortical neuron culture

Cortices from embryonic day 15 to 16 C57BL/6 mice were digested with 20 U/ml papain (Worthington, LS003126) at 37 °C for 30 min. Cortical neurons were cultured within Neurobasal medium (Gibco, 21,103–049) supplemented with 2% B27 (Gibco, 17,504–044) at 37 °C with proper density. We performed lentivirus transfection the day after plating and replaced the medium 24 h after transfection. We collected neurons at 6 or 7 days in vitro for subsequent experiments.

### Western blotting

The protein samples were harvested from cultured cortical neurons with RIPA buffer, and mouse brain cortices and lumbar spinal cords were homogenized in RIPA buffer (containing 150 mM NaCl, 1% sodium deoxycholate, 0.1% SDS, 50 mM Tris-HCl pH 7.4, 1% Triton X-100 and protease inhibitor cocktail tablets (Roche, 04693159001)). We sonicated the lysates with 10 sets of 30-s pulses on ice cold and the lysates were centrifuged at 13,000 rpm for 10 min at 4 °C. The protein samples from the supernatant were run on 8–10% SDS-PAGEs at constant voltage and then transferred to PVDF membranes (Millipore, pore size: 0.45 μm). Blots were blocked in 5% BSA in PBS-Tween for 2 h at room temperature and then incubated with primary antibodies overnight at 4 °C. After washing with PBS-Tween, the blots were incubated with secondary antibodies for 2 h at room temperature. The protein bands were detected with chemiluminescence (ECL Western Blotting Substrate, Pierce, #32106).

### Immunohistochemistry

For cultured primary neurons, we aspirated the medium and washed cells with PBS. 4% paraformaldehyde (PFA) in PBS was used to fix the cells for 20 min at room temperature. After washing with PBS, cells were incubated in block buffer (3% BSA, 0.1% Triton X-100 in PBS) for 2 h at room temperature. Then cells were incubated with primary antibodies overnight at 4 °C, followed by the incubation of secondary antibodies for 2 h at room temperature. For acquiring brain and spinal cord sections, animals were perfused transcardially with PBS then 4% PFA. After fixation in 4% PFA, brains and spinal cords were cut 40 μm thick with cryostats sectioning of Leica CM1950. After washing in PBS, sections were incubated in block buffer (5% BSA, 0.3% Triton X-100 in PBS) for 2 h at room temperature. Then sections were incubated with primary antibodies overnight at 4 °C, followed by the incubation of secondary antibodies for 2 h at room temperature. All images were captured on Nikon TiE-A1 plus confocal microscope.

### Quantitative analysis of immunohistochemistry data

When counting ChAT-positive motor neurons in lumbar spinal cords, confocal imaging would include an intact ventral horn of spinal cord which could show all motor neurons in half a section. Then we counted the number of ChAT-positive cells co-localized with DAPI. As for microglia imaging, we chose similar regions among all sections and counted Iba1-positive cells which could be discriminated easily.

During quantitative analysis of neuromuscular junctions (NMJs), postsynaptic acetylcholine receptors were labeled with α-bungarotoxin (BTX) CF488A conjugate (green), motor neuron axons were labeled with anti-neurofilament-L (NF) antibody (red), and synapses were labeled with anti-synapsin-1 (Syn) antibody (red). The co-localization of green and red signals was considered as intact or innervated NMJ. We calculated the innervation ratio per 100 NMJs and the significant decrease in this ratio reflected NMJ denervation.

### Morphological analysis

We used Simple Neurite Tracer plugin in Image J software to quantify the total axon length of cultured neurons. For microglia morphology analysis, we used polygon selection tool to surround the projection of the cell bodies of Iba1-positive channel to calculate the soma area and roundness of microglia. Then we utilized the skeleton analysis method as previously reported [31] with minor modifications to estimate the length and number of branches per cell. In brief, we firstly visualized branches as many as possible, and then de-speckled noise signals to clear the background. After binary images were made, we used Skeletonize plugin in Image J, and then applied Analyze Skeleton plugin to calculate the length and number of skeletonized branches. Activated microglia represent larger soma size, decreased soma roundness and fewer branches.

### RNA isolation and quantitative RT-PCR

Total RNA was collected from neuron cultures or mouse tissue homogenates in Trizol reagent (Invitrogen, 15,596–018) and extracted as manufacturer’s instructions. The reverse transcription was carried out with PrimeScript RT Master Mix (TaKaRa, RR036A), and 500–1000 ng total RNA was used per reaction. We performed real-time PCR with SYBR green premix (Toyobo, QPK-201) and analyzed data on the StepOnePlus Real-Time PCR System (Applied Biosystems). β-Actin was used as internal control.

### Chromatin immunoprecipitation

ChIP Assay Kit (Upstate, 17–295) was used to perform ChIP assays according to the standard protocol in cultured cortical neurons. 200 base pairs ahead of the transcriptional start site were considered as the promoter region that interesting proteins bound. The sheared DNA samples were amplified and analyzed by real-time PCR. Signals were presented as the percentage of input. Anti-CREST (Willget) and anti-HDAC1 (Abcam, ab7028) antibodies were used in this experiment. IgG was the negative control.

### Behavior tests

When CREST knockout mice and WT littermates were at the age of 12 months, rotarod tests were performed on a professional apparatus (Ugo Basile S.R.L.) every 2 weeks until 18 months. During this period, we used accelerating mode in which the rotating speed began at 8 rpm, and then elevated to 80 rpm in 300 s. At 18 months, we also performed fixed speed mode of rotarod tests on CREST knockout mice and WT littermates. In this mode, the rotating speed began at 20 rpm, and then lasted for 60 s. We also performed accelerating rotarod tests on Q394X knock-in mice at 12 or 18 months. We used two kinds of acceleration programs (4 to 40 rpm, or 8 to 80 rpm in 300 s). We performed three trials in each session and measured the time mice could hold on the rotating rod to indicate the motor function. The apparatus setup of beam walking test was produced as previously described [32]. We chose the square beam whose side length was 12 mm for training and test sessions. After three training days, we performed two consecutive trials on each mouse and recorded with video camera in the test session. We analyzed the beam traversing time and the slip times of hind paws of both CREST knockout and Q394X knock-in mice at 18 months.

In open field tests, mice were allowed to move freely in a 40 cm × 40 cm white box for 10 min. We analyzed the video recordings by the Ethovision XT software (Noldus) to measure the distance mice moved and the time mice stayed in the center region. We used CatWalk XT system (Noldus) to analyze the gait and locomotion of knockout mice. We chose a region of 20 cm length as the capture zone, and footprints could be visualized and analyzed when mice traversed this zone. The grip strength apparatus (Ugo Basile) was applied to measure the forelimb strength of knockout mice at 18 months.

### Statistical analysis

We used Graph Pad Prism 6 software (La Jolla) as the statistics tool to determine whether there were statistically significant differences between groups. Student’s t test was used in the analysis between two groups. One-way ANOVA was used between three groups. Two-way ANOVA was used in the analysis consisted of two factors. The meanings of *p* values are indicated as blow: *p < 0.05, **p < 0.01, ***p < 0.001, and ****p < 0.0001.

## Results

### The Q388X mutation decreases protein stability of CREST in vitro and in vivo

**Fig. 1:**
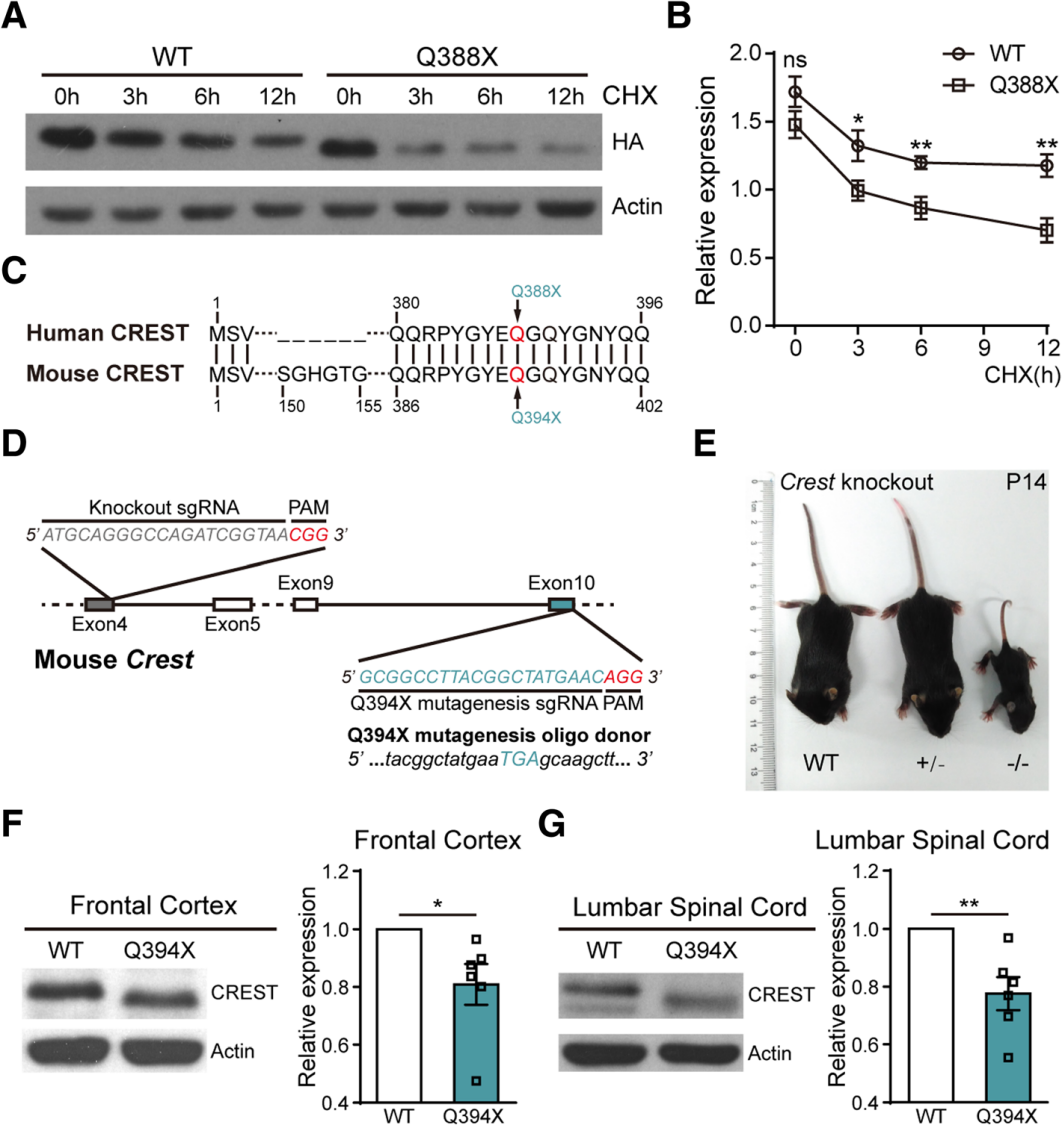
The ALS Mutation Q388X Leads to Instability of CREST Protein In Vitro and In Vivo. (**a** and **b**) Protein levels of WT and Q388X mutant form of CREST in primary cortical neurons infected with lentivirus expressing HA tagged CREST WT or Q388X cDNA respectively, treated with cycloheximide (CHX, 20μg/ml) for 0h, 3h, 6h, 12h, measured by immunoblot **a** and quantification **b** Relative expression represents the ratios of HA (upper panel) and Actin (lower panel) band intensities determined by Image J. **c** The schematic illustration for partial alignment of human CREST protein with mouse CERST protein, showing the conserved position of ALS mutation (Q388X) and the corresponding site in mouse CREST protein (Q394X). (**d**) Strategies of the constructing CREST knockout (KO) and CREST Q394X mutation mice using CRISPR/Cas9 technology. (**e**) Body size of a *Crest* ^*−/−*^ mouse compared to *Crest* ^*+/−*^ and WT littermates at P14. (f and g) Endogenous protein levels of CREST in the frontal cortices (**f**) and the lumbar spinal cords (**g**) from 6-month-old Q394X mice and WT littermates (*n* = 6, each phenotype). Error bars represent SEM. *p < 0.05, **p < 0.01, Student’s *t* test

To study protein properties of CREST-Q388X mutant, we constructed lentiviral vectors expressing cDNA of human WT CREST and Q388X mutant respectively, and infected lentivirus into primary cortical neurons isolated from embryonic C57BL/6 mice. Although there was no statistical difference between basal levels of CREST WT and Q388X mutant protein, we surprisingly found that the protein level of Q388X mutant was significantly reduced compared to WT if protein synthesis was blocked by cycloheximide (CHX) treatment, suggesting that protein stability of CREST-Q388X mutant is much lower compared to WT in cultured neurons in vitro (Fig. 1a and b).

To further examine the role of CREST Q388X mutation in vivo, we constructed CREST knockout (KO) mice and the Q394X mice carrying point mutation Q394X in the *Crest* gene (the corresponding mutation of mouse CREST homolog for human CREST-Q388X) with CRISPR/Cas9 technology (Fig. 1c and d). Consistent with previous report, most of homozygous KO (*Crest* ^*−/−*^) mice were lethal, and survivors were much smaller in size and had severe locomotion impairment compared to WT or heterozygous (*Crest* ^*+/−*^) littermates (Fig. 1e) [11]. We tested the protein level of endogenous CREST by performing Western blotting on samples collected from frontal cortices and lumbar spinal cords of Q394X and WT mice of 6 months old. Consistently, we found that protein level of CREST declined about 20% in brain and spinal cord of Q394X mice compared to WT mice, indicating that Q394X mutation leads to instability of endogenous CREST protein in vivo (Fig. 1f and g). Therefore, we suggest that CREST*-*Q388X mutant exhibits *loss-of-function* effects in causing ALS in human patients.

### Activated morphological appearance of microglia and denervation of TA muscles occur in CREST KO mice

To establish causal connection between loss of CREST and ALS pathogenesis, we set out to determine whether loss of CREST caused ALS-associated pathological features in vivo. Since homozygous *Crest* ^*−/−*^ mice could only survive less than one month, we examined histology markers in brain and lumbar spinal cord sections of *Crest* ^*−/−*^*, Crest* ^*+/−*^ and WT mice at postnatal day 14 (P14). First, we found that the number of cholinergic (ChAT-positive) motor neurons in the ventral horn of lumbar spinal cord had no statistical difference between *Crest* ^*−/−*^*, Crest* ^*+/−*^ and WT mice (Additional file 2: Figures S1A, S1B and S1C). Since inflammatory responses mediated by microglia in CNS have been implicated in numerous neurodegenerative diseases and motor neuron disorders, we would like to examine whether neuroinflammation was evoked in the absence of CREST. We measured inflammatory responses by immunostaining Iba1, the marker for microglia, in cerebral motor cortex and lumbar spinal cord of *Crest* ^*−/−*^*, Crest* ^*+/−*^ and WT mice at P14. In terms of the overall cell number of Iba1-positive microglia, we did not detect alteration in either motor cortex or lumbar spinal cord of *Crest* ^*−/−*^*,* or *Crest* ^*+/−*^ mice compared to WT littermates (Additional file 2: Figures S1D, S1E and S1F). However, we observed that Iba1-positive microglia represented activated morphological features, including enlarged cell bodies and less complex branches, in both lumbar spinal cord and motor cortex of CREST KO mice, compared to WT littermates (Additional file 2: Figures S1G-S1P). Moreover, we showed more dramatic decrease of the length and number of microglial branches in lumbar spinal cord and motor cortex of *Crest* ^*−/−*^ mice compared to *Crest* ^*+/−*^ littermates (Additional file 2: Figures S1J, S1K, S1O and S1P), validating the causality of CREST insufficiency with microglial activation. It was also worth to mention that we measured higher microglial activation level in spinal cord than in motor cortex of *Crest* ^*+/−*^ mice at P14 (Additional file 2: Figures S1J, S1K, S1O and S1P).

**Fig. 2:**
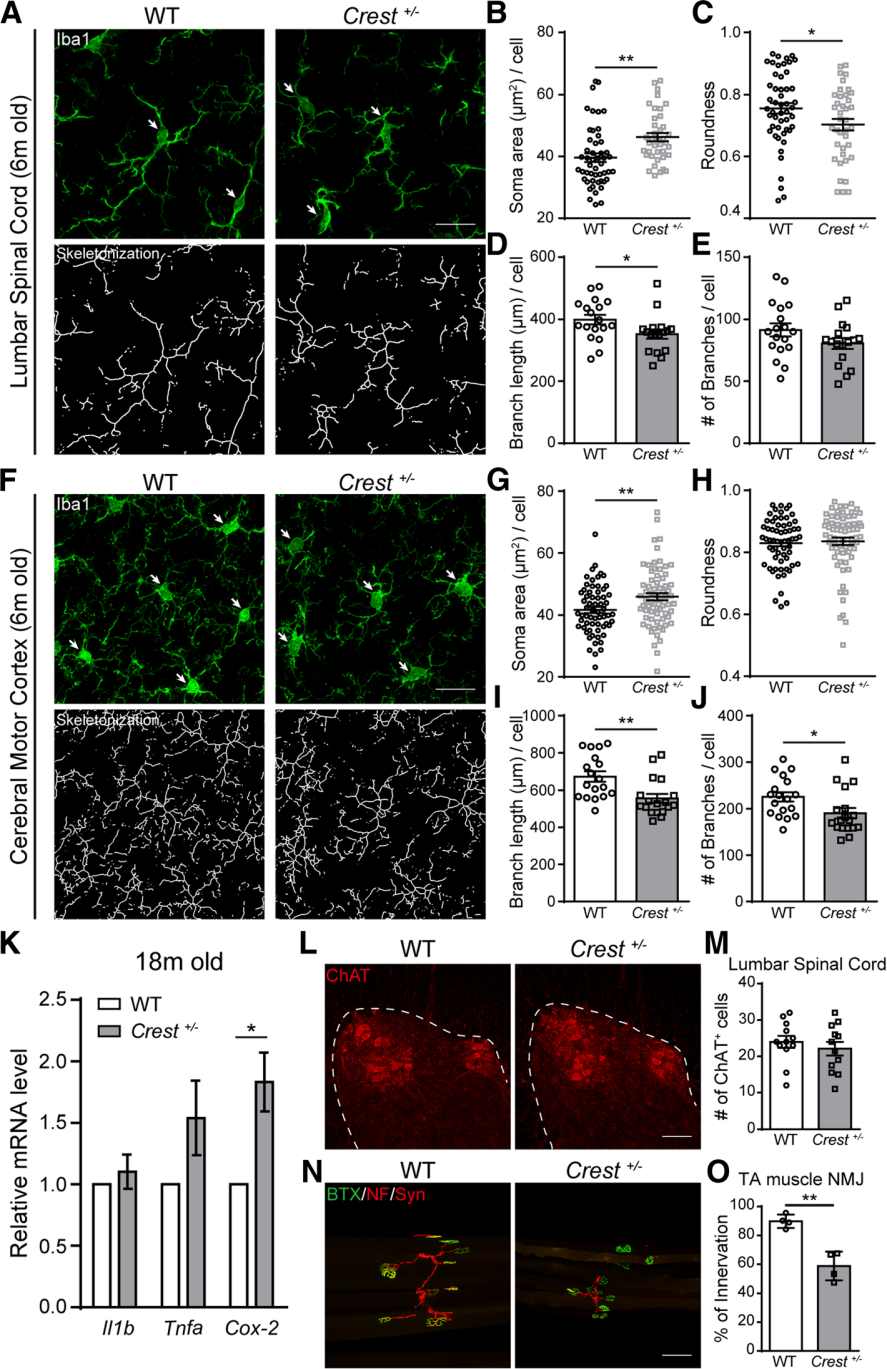
CREST Haploinsufficiency Leads to Upregulation of Inflammatory Responses in the Central Nervous System and the Denervation of Neuromuscular Junctions in TA Muscles. **a** Representative immunohistochemistry images of Iba1-positive microglia (highlighted by arrows, green, upper panels) and their skeletonized appearance (lower panels) in the lumbar spinal cords of 6-month-old *Crest* ^*+/−*^ mice (*n* = 3) and WT littermates (n = 3). Scale bar, 20μm. (**b**-**e**) Analysis and quantification of microglial morphological parameters including soma area (**b**) and roundness (**c**) of the projection of Iba1-positive cell bodies (each symbol indicating one cell), and branch length (**d**) and branch number (**e**) per cell (each symbol indicating one image, about 6 images per mouse) in the lumbar spinal cords of 6-month-old *Crest* ^*+/−*^ mice (n = 3) and WT littermates (n = 3). **f** Representative immunohistochemistry images of Iba1-positive microglia (highlighted by arrows, green, upper panels) and their skeletonized appearance (lower panels) in the cerebral motor cortices of 6-month-old *Crest* ^*+/−*^ mice (n = 3) and WT littermates (n = 3). Scale bar, 20μm. (G-J) Analysis and quantification of microglial morphological parameters including soma area (**g**) and roundness (**h**) of the projection of Iba1-positive cell bodies (each symbol indicating one cell), and branch length (**i**) and branch number (**j**) per cell (each symbol indicating one image, about 6 images per mouse) in the cerebral motor cortices of 6-month-old *Crest* ^*+/−*^ mice (n = 3) and WT littermates (n = 3). **k** mRNA levels of proinflammatory genes, such as IL-1β, TNF-α and COX-2, in frontal cortices from 18-month-old *Crest* ^*+/−*^ mice (*n* = 9) and WT littermates (n = 3) measured by quantitative RT-PCR. (**l** and **m**) Representative immunohistochemistry images (**l)** and quantification (**m**) of ChAT-positive (red) motor neurons (each symbol indicating one image, 4 images per mouse) in ventral horn of the lumbar spinal cords of 6-month-old *Crest* ^*+/−*^ mice (n = 3) and WT littermates (n = 3). Dashed lines indicate grey matter of lumbar spinal cord. Scale bar, 100μm. (**n** and **o**) Representative immunohistochemistry images (**n**) and quantification (**o**) of the innervation of neuromuscular junctions in tibialis anterior (TA) muscles of 6-month-old *Crest* ^*+/−*^ mice (n = 3) and WT littermates (n = 3). Each symbol indicates one innervation ratio of about 100 neuromuscular junctions). Acetylcholine receptors were labeled with α-bungarotoxin (BTX) CF488A conjugate (green), motor neuron axons were labeled with anti-neurofilament-L (NF) antibody (red), and synapses were labeled with anti-synapsin-1 (Syn) antibody (red). Scale bar, 100μm. Error bars represent SEM. *p < 0.05, **p < 0.01, Student’s *t* test

We further performed immunohistochemistry analysis with Iba1 antibody in aged *Crest* ^*+/−*^ and WT mice at 6 months of age. Consistently, we observed morphological features of chronically activated microglia (increased soma area and decreased branch length or number) in both lumbar spinal cord and motor cortex in *Crest* ^*+/−*^ mice, but not in WT mice of the same age (Figs. 2a-j). Interestingly, we noticed that the complexity of microglial branches did not change in cerebral motor cortex of *Crest* ^*+/−*^ mice at P14, but at 6 months, significant decreases in branch length and number were detected in the same region, suggesting that the extent of microglial activation increases in *Crest* ^*+/−*^ mice with age (Fig. 2i, j, Additional file 2: Figures S1O and S1P). Taken together, we demonstrate that loss of CREST leads to microglial activation in the CNS of mice.

Besides morphological analysis of microglia, we also tested expression levels of genes associated with neurotoxic proinflammation in *Crest* ^*+/−*^ and WT mice. Interestingly, we found a 2-fold upregulation of proinflammatory gene cyclooxygenase-2 (*Cox*-2, also known as *Ptgs2*), but not IL-1β (*Il1b*) or TNF-α (*Tnfa*), in cortical tissue of *Crest* ^*+/−*^ mice at 18 months of age (Fig. 2k). This result is consistent with previous reports showing increased expression of *Cox*-2 in both ALS patients and SOD1 mouse model [33, 34]. Together, we demonstrate that loss of CREST results in both microglial activation and the onset of proinflammatory processes in CNS.

We further analyzed neurodegenerative phenotypes of both somatic and axonal morphology of motor neurons in lumbar spinal cord of *Crest* ^*+/−*^ mice and WT littermates at 6 months of age. Although there was no statistical difference in the number of ChAT-positive motor neurons in lumbar ventral horn between WT and *Crest* ^*+/−*^ mice (Fig. 2l and m), we found that the ratio of innervation of neuromuscular junctions (NMJs) in TA muscles significantly decreased in *Crest* ^*+/−*^ mice compared to WT littermates (Fig. 2n and o), suggesting a sign of axonal degeneration of motor neurons in lumbar ventral horn of *Crest* ^*+/−*^ mice. Moreover, we intend to determine whether loss of CREST directly leads to impaired axonal morphology in a cell-autonomous manner by analyzing some pathological features in primary cultured neurons in vitro. As an important hallmark of ALS, aberrant accumulation of protein inclusions in cellular cytoplasm may be toxic to neurons [35–37]. However, we did not find that the formation of YB1-positive stress granules under the treatment of sodium arsenite was influenced in isolated primary cortical neurons from embryonic CREST KO or WT littermates (Additional file 3: Figures S2A and S2B). To further illustrate dosage effects of CREST on axonal morphology, we transfected plasmids expressing short hairpin RNA (shRNA) of CREST [12] or human WT CREST cDNA into mouse primary cultured neurons and detected the axonal phenotype by immunostaining with axonal marker SMI312. We found that neither knockdown nor overexpression of CREST in neurons affected axonal length (Additional file 3: Figures S2C and S2D). Taken together, our results suggest that CREST deficiency may not cause cell-autonomous toxicity to neurons, and the denervation of NMJs may be caused by chronic microglial activation, proinflammatory responses or other causes.

### Activation of microglia occurs in CREST Q394X mice

**Fig. 3:**
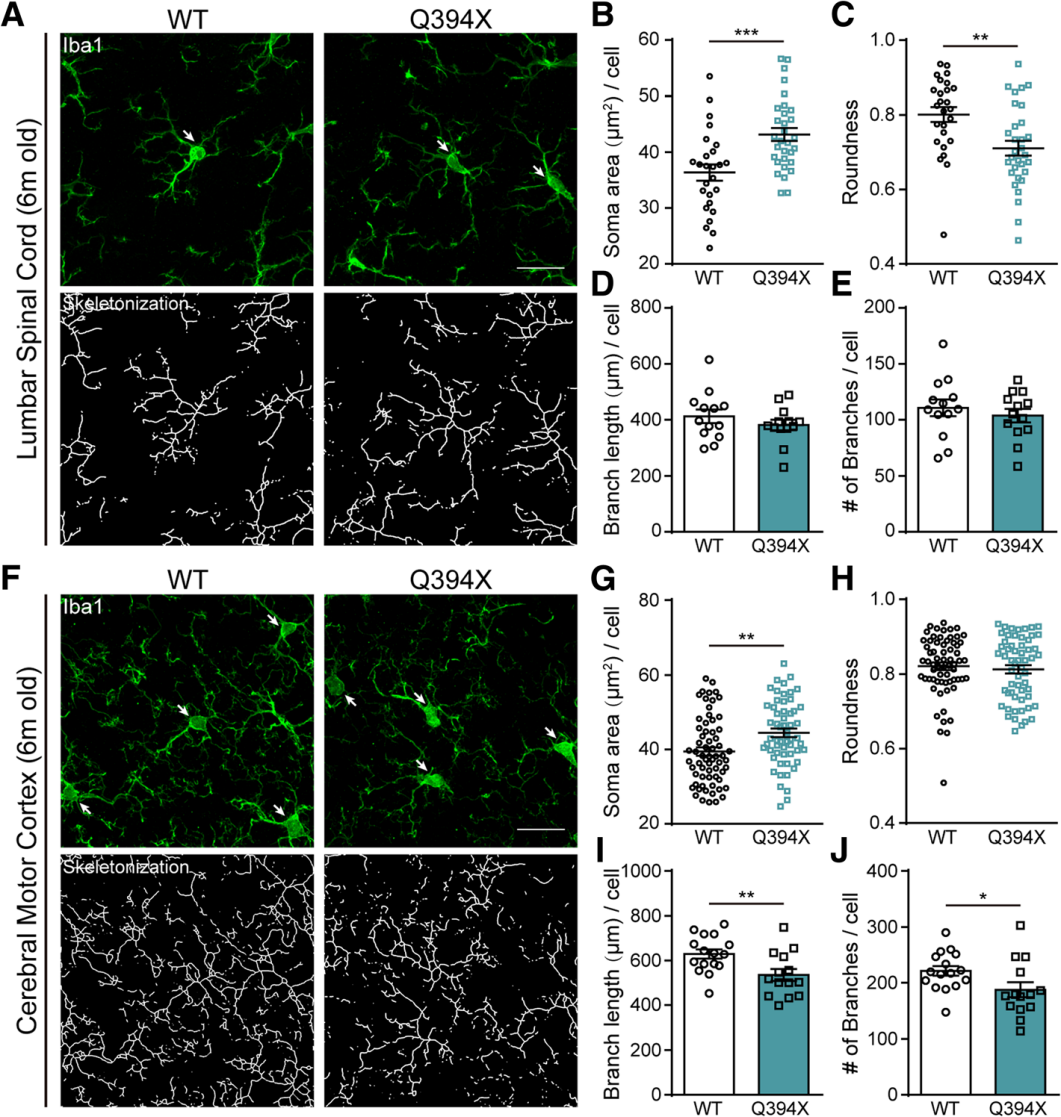
Microglia Are Prevalently Activated in Q394X Mice. **a** Representative immunohistochemistry images of Iba1-positive microglia (highlighted with arrows, green, upper panels) and their skeletonized appearance (lower panels) in the lumbar spinal cords of 6-month-old Q394X mice (*n* = 3) and WT littermates (n = 3). Scale bar, 20μm. (**b**-**e**) Analysis and quantification of microglial morphological parameters including soma area (**b**) and roundness (**c**) of the projection of Iba1-positive cell bodies (each symbol indicating one cell), and branch length (**d**) and branch number (**e**) per cell (each symbol indicating one image, about 5 images per mouse) in the lumbar spinal cords of 6-month-old Q394X mice (n = 3) and WT littermates (n = 3). (**f**) Representative immunohistochemistry images of Iba1-positive microglia (highlighted with arrows, green, upper panels) and their skeletonized appearance (lower panels) in the cerebral motor cortices of 6-month-old Q394X mice (n = 3) and WT littermates (n = 3). Scale bar, 20μm. (g-j) Analysis and quantification of microglial morphological parameters including soma area (**g**) and roundness (**h**) of the projection of Iba1-positive cell bodies (each symbol indicating one cell), and branch length (**i**) and branch number (**j**) per cell (each symbol indicating one image, about 5 images per mouse) in the cerebral motor cortices of 6-month-old Q394X mice (n = 3) and WT littermates (n = 3). Error bars represent SEM. *p < 0.05, **p < 0.01, and ***p < 0.001, Student’s *t* test

Next, we would like to determine whether inflammatory responses are also activated in CNS of Q394X mutant mice. After immunohistochemical analysis of Iba1-positive microglia in cerebral motor cortex and lumbar spinal cord of Q394X mice and WT littermates, we showed significant increase in soma area and decrease in soma roundness but no alteration of branch complexity of microglia in lumbar spinal cord of Q394X mice at 6 months, compared to WT littermates (Figs. 3a-e). Moreover, there were increase of soma area and decrease of length and number of branches of microglia in motor cortex of Q394X mice compared to WT at 6 months (Figs. 3f-j). Therefore, we identified similar signs of increased inflammatory responses in motor cortex and lumbar spinal cord of Q394X mice, as they are in CREST haplosufficiency mice, suggesting that Q394X mice exhibits neuropathological symptoms in CNS.

### CD68 signals increase in CREST KO and Q394X mice

**Fig. 4:**
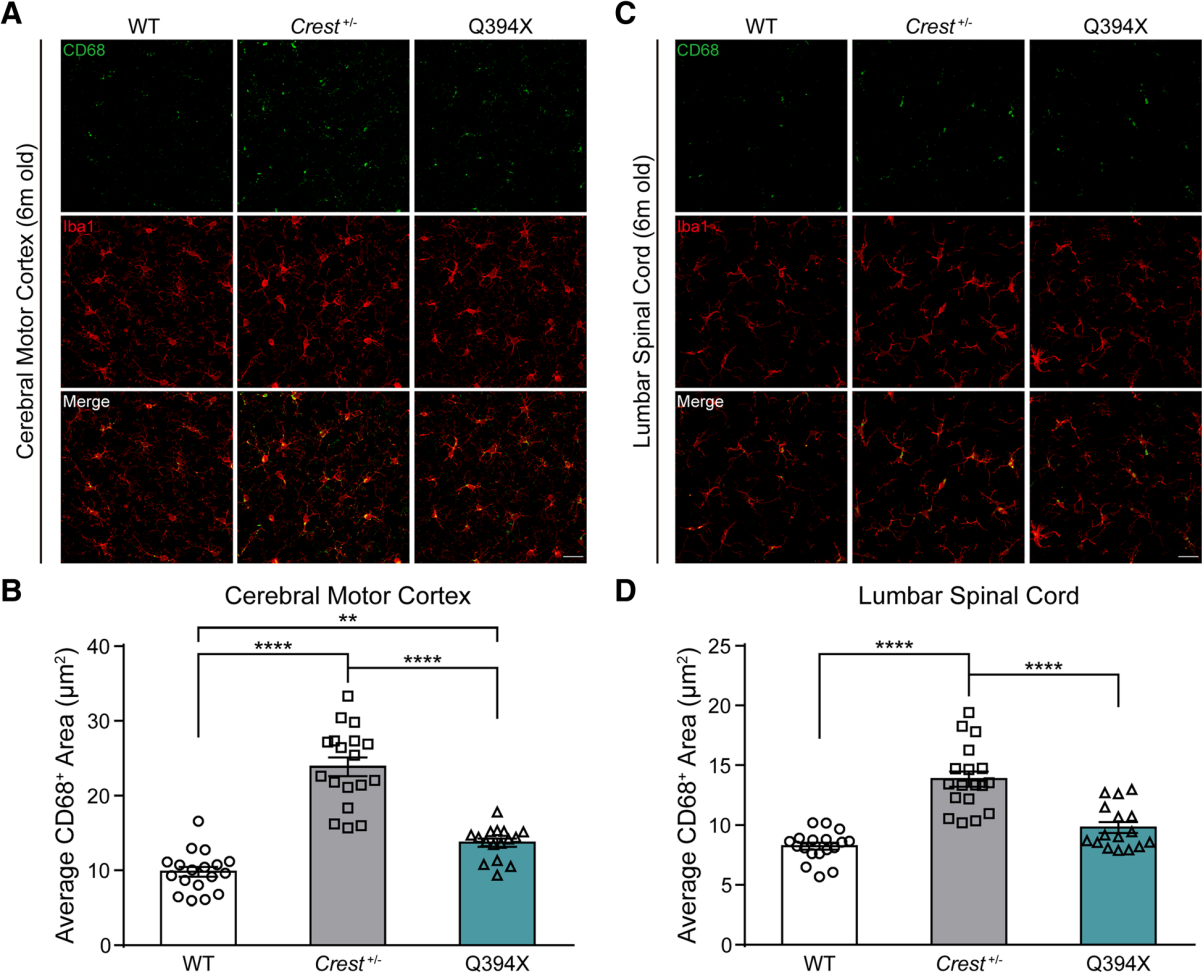
CD68 Staining Confirms Microglial Activation in CREST mutant Mice. (**a** and **b**) Representative immunohistochemistry images (**a**) and quantification (**b**) of CD68 (green) and Iba1 (red) cells in the cerebral motor cortices of 6-month-old *Crest* ^*+/−*^ mice (n = 3), 6-month-old Q394X mice (n = 3) and WT mice (n = 3). Each symbol indicates one image, about 5 or 6 images per mouse. Scale bar, 25μm. (C and D) Representative immunohistochemistry images (**c**) and quantification (**d**) of CD68 (green) and Iba1 (red) cells in the lumbar spinal cords of 6-month-old *Crest* ^*+/−*^ mice (n = 3), 6-month-old Q394X mice (n = 3) and WT mice (n = 3). Each symbol indicates one image, about 5 or 6 images per mouse. Scale bar, 25μm. Error bars represent SEM. **p < 0.01 and ****p < 0.0001, one-way ANOVA

Besides morphological features indicating microglial activation, we would like to further validate the finding by examining the signal of CD68, which is a typical biomarker of activated macrophages, including microglia. After performing immunohistochemical analysis of CD68 signal area per microglia, we found that CD68 signals significantly increased in cerebral motor cortex of both *Crest* ^*+/−*^ and Q394X mice compared to WT at 6 months (Fig. 4a and b). As for lumbar spinal cord, we observed distinct upregulation of CD68 in *Crest* ^*+/−*^ mice and a mild but not significant increase of CD68 staining in Q394X mice compared to WT at 6 months (Fig. 4c and d). Furthermore, there were more CD68 signals both in cerebral motor cortex and lumbar spinal cord of *Crest* ^*+/−*^ mice compared to Q394X mice (Figs. 4a-d). Thus, we confirmed microglial activation both in *Crest* ^*+/−*^ and Q394X mice by CD68 staining, and consistent with the extent of CREST deficiency, *Crest* ^*+/−*^ mice have more activated microglia compared to Q394X mice.

### Both CREST KO and Q394X mice show the impairment of motor phenotypes

**Fig. 5:**
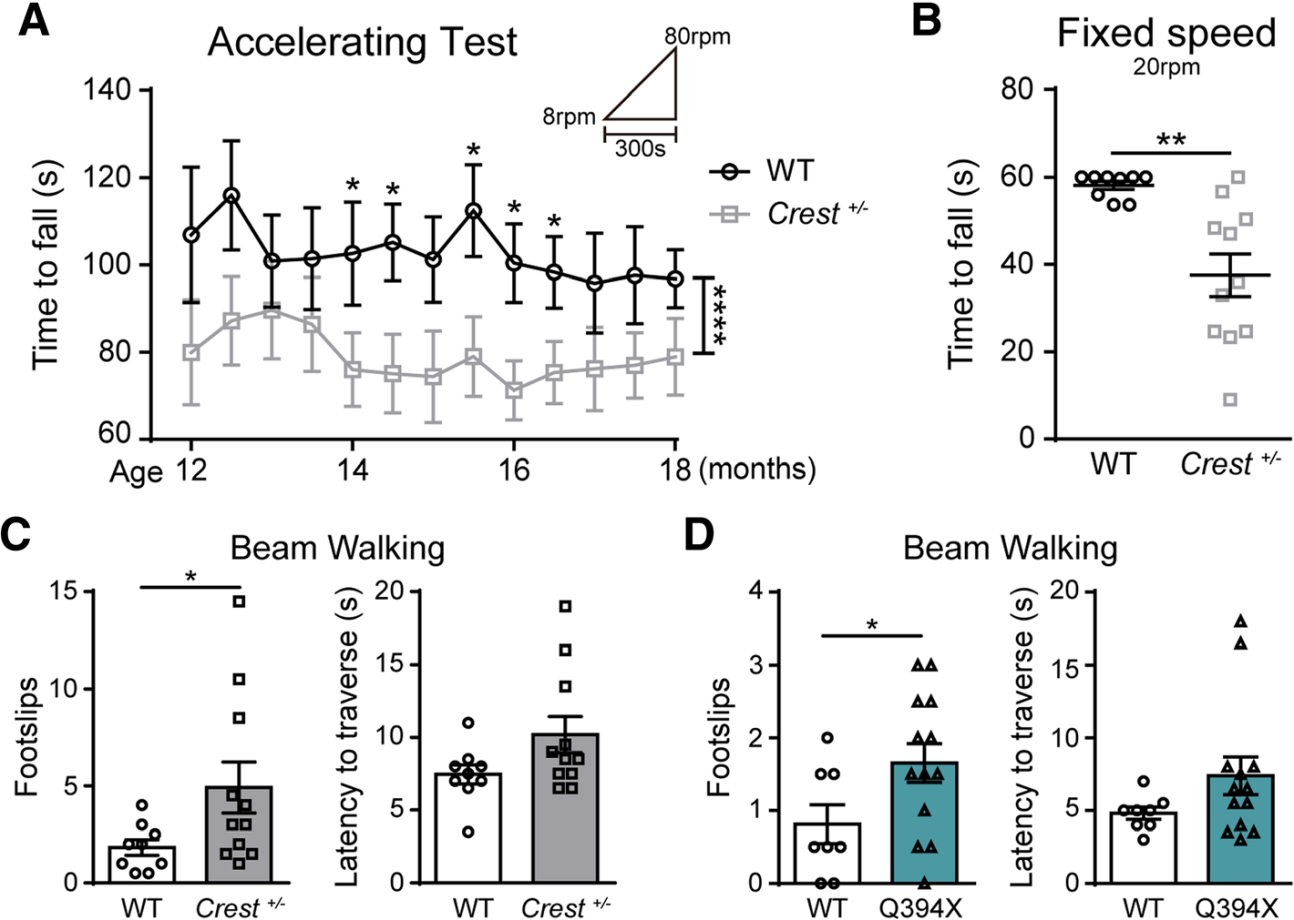
Both *CREST* ^*+/−*^ and Q394X Mice Display Impaired Motor Coordination. **a** Behavioral performance of *Crest* ^*+/−*^ mice (*n* = 11) and WT littermates (n = 9) in accelerating mode of rotarod tests from 8 rpm to 80 rpm within 300 s from the age of 12 months to 18 months. Tests were performed every two weeks. **b** Performance of *Crest* ^*+/−*^ mice (n = 11) and WT littermates (n = 9) in fixed speed mode of rotarod tests at 20 rpm up to 60 s at the age of 18 months. (C and D) The beam walking tests with square beam on *Crest* ^*+/−*^ mice (n = 11) and WT littermates (n = 9) (**c**), and on Q394X mice (*n* = 13) and WT littermates (*n* = 8) (**d**) at 18 months of age. The performance was measured by times of hind paw slips (left panel) and the latency to traverse the beam (right panel). Error bars represent SEM. *p < 0.05, **p < 0.01, Student’s *t* test. ****p < 0.0001, two-way ANOVA


To determine whether *Crest* ^*+/−*^ or Q394X mice may exhibit ALS-like motor defects, we performed a battery of behavioral tests including open field, footprint, grip strength, rotarod and beam walking [38] on *Crest* ^*+/−*^ mice and WT littermates. *Crest* ^*+/−*^ mice appeared to be generally healthy during 12 to 18 months of age (Additional file 4: Figure S3A), and exhibited no defects in open field test (Additional file 4: Figure S3B). Although we observed increased width of front foot bases (Additional file 4: Figure S3C) and decreased forelimb strength (Additional file 4: Figure S3D) of *Crest* ^*+/−*^ mice at 18 months, the difference was not significant enough in footprint and grip strength tests (Additional file 4: Figures S3C and S3D). However, we found that *Crest* ^*+/−*^ mice started to exhibit deficits of motor coordination in the accelerating rotarod test from 14 months of age (Fig. 5a). Moreover, *Crest* ^*+/−*^ mice fell more easily from rotating rod than WT littermates in the fixed speed task of rotarod test (Fig. 5b). In beam walking test, *Crest* ^*+/−*^ mice showed significantly more slipped steps compared to WT mice (Fig. 5c, Additional file 5: Video S1 and Additional file 6: Video S2), indicating that motor coordination abilities of *Crest* ^*+/−*^ mice are indeed compromised.

**Additional file 5: Video S1.** Example trial of WT mouse (as the control of *Crest* ^*+/−*^ mouse) in beam walking test. (MP4 6693 kb)



**Additional file 6**: **Video S2.** Example trial of *Crest* ^*+/−*^ mouse in beam walking test. (MP4 7.2 mb). (MP4 7321 kb)


We further examined motor phenotypes of Q394X mice at 18 months of age. The general body weight and health of Q394X mice were also normal (Additional file 7: Figure S4A). Although exhibiting no defects in open field and rotarod tests (Additional file 7: Figures. S4B and S4C), Q394X mice also showed more foot slips in beam walking test compared to WT littermates (Fig. 5d, Additional file 8: Video S3 and Additional file 9: Video S4), indicating similar impairment of motor phenotypes in Q394X mice as in *Crest* ^*+/−*^ mice. Taken together, we demonstrate that both haploinsufficiency and Q394X mutation of CREST can lead to elevated inflammatory responses in CNS and deficits in motor coordination in vivo.


**Additional file 8**: **Video S3.** Example trial of WT mouse (as the control of Q394X mouse) in beam walking test. (MP4 4839 kb)



**Additional file 9**: **Video S4.** Example trial of Q394X mouse in beam walking test. (MP4 8580 kb)


### The CREST-BRG1 complex suppresses expression of cytokine genes *Ccl2* and *Cxcl10* via histone deacetylation in neurons

Prior to the investigation of molecular mechanisms underlying increased inflammatory responses in CREST deficient mice, we sought to determine expression pattern of CREST in CNS. According to the data from Brain RNA-Seq database [39] and previous report, *CREST* is highly expressed in neurons compared to other cell types in both human and mouse CNS (Additional file 10: Figure S5A) [11]. We confirmed this conclusion by performing immunohistochemistry experiments with various antibodies in cerebral motor cortex and lumbar spinal cord of adult WT mice. In agreement with previous data, we found that CREST mostly was not expressed in Iba1-positive microglia either in motor cortex or lumbar spinal cord (Additional file 10: Figures S5B and S5C). However, CREST showed some expression in GFAP-positive astrocytes in white matter of lumbar spinal cord but not in motor cortex of WT mice (Additional file 10: Figures S5B and S5C). Therefore, we suggest that neuronal loss of CREST may contribute to the microglial activation in CNS.

**Fig. 6:**
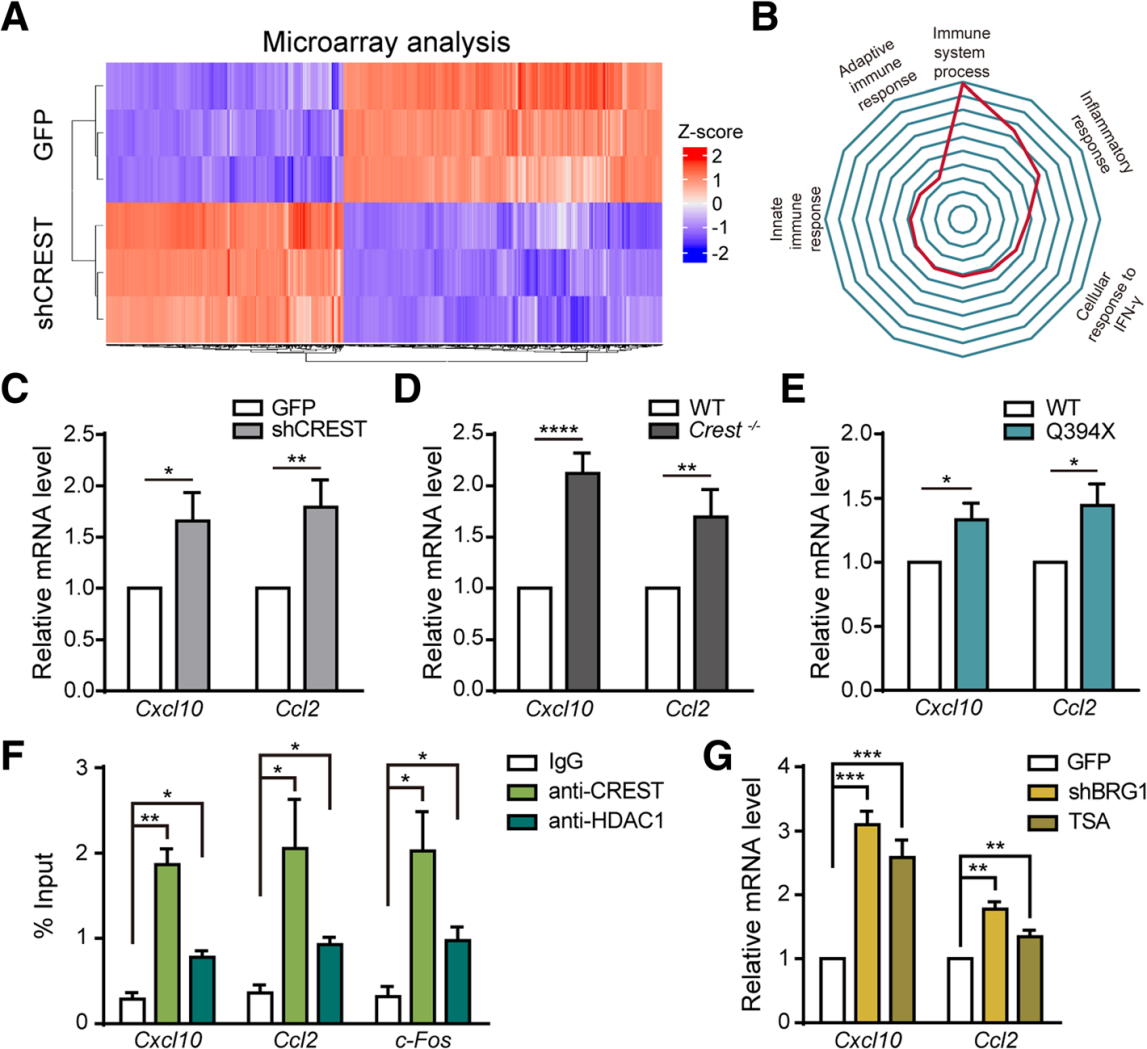
The CREST-BRG1 Complex Represses the Expression of Chemokine Genes via Recruiting Histone Deacetylation Complex in Neurons. **a** Heatmap showing the differentially expressed genes in primary cortical neurons infected with lentivirus expressing short hairpin RNA targeting CREST (shCREST) or expressing GFP (as control) by microarray analysis (fold change over 2.0). **b** Radar map showing the gene ontology analysis of gene lists in (A). **c** Quantitative RT-PCR of two chemokine genes, *Cxcl10* and *Ccl2*, for the confirmation of microarray data in primary cortical neurons transfected with shCREST or GFP as control by lentivirus. (D and E) Quantitative RT-PCR experiments validating the relative mRNA levels of *Cxcl10* and *Ccl2* in primary cortical neurons isolated from embryotic *Crest* ^*−/−*^ **d** and Q394X **e** mice compared to their WT littermates, respectively. **f** Chromatin immunoprecipitation on promoter regions of *Cxcl10*, *Ccl2* and *c-Fos* genes with IgG, anti-CREST or anti-HDAC1 antibody in primary cortical neurons. Signals were normalized as percentage of input. **g** Quantitative RT-PCR of *Cxcl10* and *Ccl2* in primary cortical neurons treated with 0.5μM trichostatin A (TSA) for 6h, or infected by lentivirus expressing shRNA targeting BRG1 (shBRG1) or GFP (as control). Error bars represent SEM. *p < 0.05, **p < 0.01, ***p < 0.001, and ****p < 0.0001, Student’s *t* test

Given the fact that CREST is a critical transcriptional regulator [12], we examined gene expression profiles by performing microarray analysis in primary cortical neurons infected with lentivirus harboring shRNA targeting CREST or control shRNA (Fig. 6a and Additional file 11: Figure S6B). Using DAVID bioinformatics resources online (https://david.ncifcrf.gov) [40, 41], we performed gene ontology (GO) analysis of microarray data and surprisingly found that most significantly affected genes were predominantly associated with immune-associated processes (Fig. 6b), suggesting the role of CREST in regulating expression of genes related to inflammatory responses. Among top 10 of the most remarkably upregulated genes, we selected two important chemokines, *Ccl2* and *Cxcl10*, which have been reported to play critical roles in microglial activation and inflammatory responses, for further analysis [42, 43]. We first confirmed that mRNA levels of *Ccl2* and *Cxcl10* were significantly upregulated in CREST knockdown neurons compared to control neurons with real-time PCR (Fig. 6c). Moreover, we observed a significant decrease in mRNA level of *Cxcl10* in CREST overexpressing neurons (Additional file 11: Figures S6C and S6D). We then examined expression levels of endogenous *Ccl2* and *Cxcl10* in primary cortical neurons isolated from embryonic cortices of *Crest* ^*−/−*^ (Additional file 11: Figure S6A), Q394X and WT mice, respectively. Importantly, we found that mRNA levels of both *Ccl2* and *Cxcl10* were markedly higher in *Crest* ^*−/−*^ and Q394X neurons compared to WT littermates, respectively (Fig. 6d and e). Thus, we demonstrate that neuronal loss of CREST leads to altered expression of immune-related genes, such as the upregulation of chemokines *Ccl2* and *Cxcl10*, which may contribute to microglial activation and elevated inflammatory responses.

To further reveal molecular mechanism underlying the regulation of *Ccl2* and *Cxcl10* in neurons by CREST, we hypothesized that CREST inhibited transcription of these two genes through the BRG1-CREST complex recruiting histone deacetylases such as HDAC1 [12]. To test this hypothesis, we performed chromatin immunoprecipitation (ChIP) experiments in cultured neurons with anti-CREST and anti-HDAC1 antibodies. Indeed, we found that both CREST and HDAC1 interacted with promoter regions of *Ccl2* and *Cxcl10* genes (Fig. 6f). Moreover, mRNA levels of *Ccl2* and *Cxcl10* were increased in BRG1 knockdown cortical neurons by lentiviral shRNA transfection (Additional file 11: Figures S6E and S6F) compared to control neurons (Fig. 6g). Consistently, we detected significantly upregulated mRNA levels of *Ccl2* and *Cxcl10* in neurons treated with an HDAC inhibitor trichostatin A (TSA) compared to controls (Fig. 6g). Taken together, we suggest that the CREST-BRG1 complex plays a critical role in inhibiting transcription of *Ccl2* and *Cxcl10* in neurons via HDAC-dependent histone deacetylation.

### Decreased protein level of FUS in CNS of CREST KO mice

**Fig. 7:**
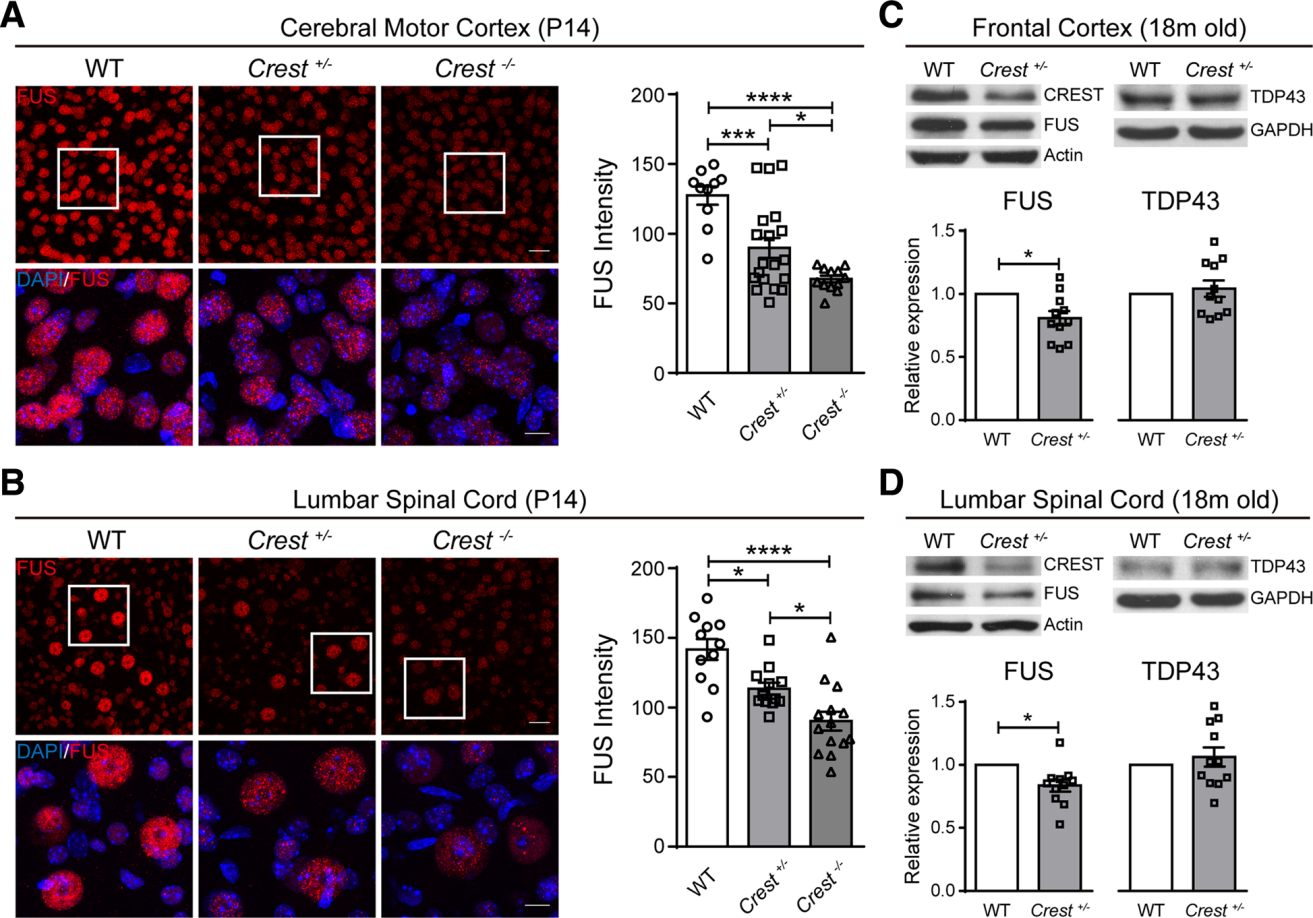
CREST and FUS Expression Represent Potentially Positive Correlation in CNS (A and B) Representative confocal immunohistochemistry images (left panels) and intensity quantification (right graphs) of FUS (red) in the cerebral motor cortices (**a**) and the lumbar spinal cords (**b**) of *Crest* ^*−/−*^ (n = 3), *Crest* ^*+/−*^ mice (n = 3) and WT littermates (n = 3) at P14. Each symbol indicates one image. Top panels show lower magnification, scale bar = 25 μm. Bottom panels show higher magnification of the white square regions of upper panels, scale bar = 10 μm. Error bars represent SEM. *p < 0.05, ***p < 0.001, and ****p < 0.0001, one-way ANOVA. (C and D) Immunoblot (top panels) and intensity quantification (bottom graphs) of protein from the frontal cortices (**c**) and the lumbar spinal cords (**d**) of *Crest* ^*+/−*^ mice (n = 11) and WT littermates (*n* = 5) at 18 months of age. Relative expression represents the normalized ratios of FUS/Actin (bottom left graphs) or of TDP43/GAPDH (bottom right graphs) band intensities determined by densitometry in Image J. Error bars represent SEM. *p < 0.05, Student’s *t* test.

Besides the regulation of immune-related genes by CREST in neurons, we also wonder whether CREST can influence the expression of other important ALS-linked genes, such as FUS which has been reported as a CREST-binding protein [8] and TDP43. We performed immunohistochemistry with anti-FUS antibody in cerebral motor cortex and lumbar spinal cord sections of *Crest* ^*−/−*^, *Crest* ^*+/−*^ and WT littermates at P14. Interestingly, we found that protein level of FUS was stringently correlated with CREST, and gradually decreased in both motor cortex and lumbar spinal cord of *Crest* ^*+/−*^ and *Crest* ^*−/−*^ mice compared to WT littermates (Fig. 7a and b). Furthermore, we found that protein level of FUS rather than TDP43 was modestly decreased both in frontal cortices and lumbar spinal cords collected from *Crest* ^*+/−*^ mice compared to WT littermates at 18 months of age (Fig. 7c and d). These data reveal that FUS may be *loss-of-function* in CREST mutant mice, further suggesting a converged pathway for ALS pathogenesis from different genetic causes.

## Discussion

Numerous publications have illustrated that chronic proinflammatory responses contribute to pathogenic mechanisms underlying the progression of neurodegenerative diseases. In this study, we reveal that microglial appearance exhibits activation morphology including the enlargement of cell bodies and the decreased complexity of branches in both CREST KO and Q394X mutant mice. Since the differentiated subsets of activated microglia, including proinflammatory (M1) and antiinflammatory (M2) microglia [44], we detected the expression of some inflammatory genes in *Crest* ^*+/−*^ mice and WT littermates at 18 months and found a specific upregulation of proinflammatory gene *Cox-2,* suggesting overall effects of CREST haploinsufficiency on chronic activation of neurotoxic M1 microglia, which may cause the axonal degeneration of motor neurons in spinal cord. Although the increased expression of *Cox-2* in ALS patients and SOD1 mutant mice has been reported [33], one more interesting discovery is that the expression of *Cox-2* is specifically increased in ALS patients rather than other neurodegenerative disease cases [34]. Moreover, inhibition of enzymatic activity of COX-2 may delay disease onset and ameliorate the survival of ALS mice [45, 46], suggesting that chronic activation of M1 microglia and concomitant upregulation of *Cox-2* caused by loss of CREST contribute to ALS pathogenesis.

Consistent with axonal degeneration in CREST haploinsufficiency mice, we found that both *Crest* ^*+/−*^ and Q394X mice exhibited deficits in motor coordination tasks, further confirming the connection between CREST and ALS pathogenesis. However, we did not detect very serious neurodegenerative phenotypes as in SOD1 mutant transgenic mice. Considering the involvement of both genetic and environmental factors in pathogenesis of ALS, we may try some artificial manipulations, such as the administration of lipopolysaccharide (LPS), in CREST KO or mutant mice to induce more significant inflammatory responses and anticipate the appearance of more typical ALS symptoms [47].

Microarray analysis suggests the altered expression of many immune-related genes, such as upregulation of chemokines *Cxcl10* and *Ccl2*, in CREST deficient or ALS-related mutation Q388X-expressing neurons. Moreover, we demonstrate that CREST may directly inhibit the transcription of *Cxcl10* and *Ccl2* genes through the CREST-BRG1-HDAC1 complex interacting with promoter regions [12]. Comprehensive regulation of immune-associated genes by CREST may play critical roles in microglial activation and proinflammatory responses, and the upregulation of chemokine genes is part of these transcriptional alterations. Consistent with the notion that upregulation of chemokines in CNS may contribute to the infiltration of periphery immune cells, increased expression of *Ccl2* in SOD1 mutant transgenic mice or ALS patients has been reported to induce the infiltration of dendritic cells and promote the acquisition of properties of antigen-presenting cells by microglia [48–50]. In this sense, it may be the involvement of multiple immune processes, possibly including T cell infiltration, that contributes to microglial activation and proinflammatory responses in CREST mutant mice.

Another interesting pathological phenotype we observed in CREST KO mice is the decreased protein level of FUS in CNS, although the reduction is quite modest. Previous publications have shown that knockdown of FUS can reduce cell viability in vitro, and cause anatomical loss of neuromuscular junctions and concomitant impairment of locomotion ability in Drosophila and zebrafish models [51–53], suggesting that besides neurotoxic proinflammation induced by microglial activation, the loss of FUS may also contribute to the degeneration of motor neurons in CREST KO mice.

## Conclusions

We demonstrate that *CREST* is a pathogenic gene for ALS, mutations of which lead to upregulation of chemokine genes in neurons, subsequently activate microglia, increase proinflammatory responses in vivo, and ultimately impair motor function in aged CREST KO and Q394X mutant mice. These data provide a transcriptional pathway for neuroinflammation activation in ALS, suggesting that approaches of modulating histone deacetylase complex may be the candidates for therapeutic intervenes.

## Additional files

Additional file 1: **Table S1.** Key Resources Table. (DOCX 74 kb)

Additional file 2: CREST KO Mice Show the Activation of Microglia in CNS at P14, related to Fig. 2. (A) Representative confocal immunohistochemistry images of CREST-positive cells (green) and ChAT-positive motor neurons (red) in the lumbar spinal cords. Dashed lines divide the white and grey matter of lumbar spinal cord. Scale bar, 100μm. (B and C) Quantification of CREST intensities (B) and the number of ChAT-positive motor neurons (C) in the lumbar spinal cords. Each symbol indicates one image. (D) Representative confocal immunohistochemistry images of Iba1-positive microglia (green) in cerebral motor cortices (left panels) and in lumbar spinal cords (right panels). Dashed lines show the lumbar spinal cords. Scale bar, 100μm. (E and F) Quantification of Iba1-positive microglia (green) in cerebral motor cortices (E) and in lumbar spinal cords (F). Each symbol indicates one image. (G) Representative immunohistochemistry images of Iba1-positive (green; arrows) microglia (top panels) and their skeletonized appearance (bottom panels) in the lumbar spinal cords. Scale bar, 20μm. (H-K) Quantification of microglial morphological parameters including soma area (H) and roundness (I) of the projection of Iba1-positive cell bodies (each symbol indicating one cell), and branch length (J) and branch number (K) per cell (each symbol indicating one image, about 6 images per mouse) in the lumbar spinal cords. (L) Representative immunohistochemistry images of Iba1-positive (green; arrows) microglia (top panels) and their skeletonized appearance (bottom panels) in the cerebral motor cortices. Scale bar, 20μm. (M-P) Quantification of microglial morphological parameters including soma area (M) and roundness (N) of the projection of Iba1-positive cell bodies (each symbol indicating one cell), and branch length (O) and branch number (P) per cell (each symbol indicating one image, about 6 images per mouse) in the cerebral motor cortices. Error bars represent SEM. *p < 0.05, **p < 0.01, ***p < 0.001, and ****p < 0.0001, one-way ANOVA. (TIF 27353 kb)

Additional file 3: **Figure S2.** Loss of CREST Function Does not Affect the Formation of Stress Granules and the Axon Length in Cultured Neurons in vitro, related to Fig. 2. (A) Representative Immunofluorescence staining of stress granule marker YB1 (red) in the treatment of nothing as control (left) or 50μM sodium arsenite (SA) for 1h (right). Arrows indicate the cells without stress granules (SGs) in the treatment of SA. Arrowheads indicate the cells with SGs. Scale bar, 15μm. (B) Quantification of the fraction of cells with SGs in cultured neurons isolated from embryotic *Crest* ^*−/−*^, *Crest* ^*+/−*^ mice and WT littermates. (C and D) Representative Immunofluorescence images (C) and the total length quantification (D) of SMI312-positive (red) axons in cultured neurons expressing GFP (C, top), shCREST labeled by GFP (C, middle) and WT human CREST labeled by GFP (green) (C, bottom). Scale bar, 100μm. Error bars represent SEM. One-way ANOVA. (TIF 13203 kb)

Additional file 4: **Figure S3.** Behavioral Tests of Motor Phenotypes on *Crest* ^*+/−*^ Mice, related to Fig. 4. (A) Body weight of *Crest* ^*+/−*^ mice (*n* = 11) and WT littermates (*n* = 9) from the age of 12 months to 18 months. The measurement was performed every 2 weeks. (B) Representative traces (two left panels), the moving distance (middle graph) and the center staying time (right graph) of *Crest* ^*+/−*^ mice (n = 11) and WT littermates (n = 9) at 18 months in open field tests. The duration of each trial was 10 min. (C) Footprint tests showing the width of support bases of both front paws (left graph) and hind paws (middle graph), and the length of strides of four paws (right graph) of *Crest* ^*+/−*^ mice (n = 11) and WT littermates (n = 9) at 18 months. (D) Grip strength tests showing the forelimb strength of *Crest* ^*+/−*^ mice (n = 11) and WT littermates (n = 9) at 18 months. Error bars represent SEM. Student’s *t* test. (TIF 8574 kb)

Additional file 7: **Figure S4.** Behavioral Tests of Motor Phenotypes on Q394X Mice, related to Fig. 4. (A) Body weight of Q394X mice (*n* = 13) and WT littermates (*n* = 8) at 18 months. (B) Open field tests showing the moving distance (left graph) and the center staying time (right graph) of Q394X mice (n = 13) and WT littermates (n = 8) at 18 months. The duration of each trial was 10 min. (C) Rotarod tests performed with two accelerating modes (4 to 40 rpm and 8 to 80 rpm in 300 s) for 3 trials on Q394X mice (n = 13) and WT littermates (n = 8) at the age of 12 months (two left graphs) and 18 months (two right graphs). Error bars represent SEM. Student’s *t* test. (TIF 12255 kb)

Additional file 10: **Figure S5.** Expression Pattern of CREST in CNS, related to Fig. 5. (A) Relative expression of CREST in cell types of human (left) and mouse (right) brain revealed by Brain RNA-Seq database. (B and C) Representative confocal immunohistochemistry images of co-localization of astrocyte marker GFAP (red) and CREST (green) (four left panels), and of microglial marker Iba1 (green) and CREST (red) (four right panels) in both cerebral motor cortex (B) and lumbar spinal cord (C). Arrows indicate GFAP-positive astrocytes or Iba1-positive microglia. Scale bar, 10μm. (TIF 21670 kb)

Additional file 11: **Figure S6.** Validation of Knockdown Efficiency of shRNAs and Inhibitory Effect of CREST on Transcription of *Cxcl10* in Cultured Neurons, related to Fig. 5. (A) Immunoblot of protein samples from primary cortical neurons isolated from embryotic *Crest* ^*−/−*^ and WT littermates, using anti-CREST (top) and anti-Actin (bottom) antibodies. (B) Immunoblot and intensity quantification of protein from primary cortical neurons infected with lentivirus carrying CREST shRNA (shCREST) and GFP (as control). Relative expression represents the intensity ratios of CREST/GAPDH. (C) Immunoblot of protein from primary cortical neurons infected with lentivirus expressing GFP or HA-tagged WT CREST, using anti-HA (top) and anti-GAPDH (bottom) antibodies. (D) Quantitative RT-PCR of *Cxcl10* (left) and *Ccl2* (right) in primary cortical neurons infected with lentivirus expressing GFP or HA-tagged WT CREST. (E and F) Immunoblot (E) and quantification (F) of protein from primary cortical neurons infected with lentivirus carrying BRG1 shRNA (shBRG1) and GFP (as control). Relative expression represents the intensity ratios of BRG1/Actin normalized to one control sample. Error bars represent SEM. *p < 0.05, ***p < 0.001, and ****p < 0.0001, Student’s *t* test. (TIF 8284 kb)

## Abbreviations

ALS: Amyotrophic lateral sclerosis
BSA: Bovine serum albumin
BTX: α-bungarotoxin
CBP: CREB-binding protein
CCL2: C-C motif chemokine ligand 2
ChAT: Choline o-acetyltransferase
ChIP: Chromatin Immunoprecipitatio
CHX: Cycloheximide
CNS: Central nervous system
COX-2: Cyclooxygenase-2
CREST: Calcium-responsive transactivator
CXCL10: C-X-C motif chemokine ligand 10
FUS: Fused in sarcoma
GAPDH: Glyceraldehyde-3-phosphate dehydrogenase
GFAP: Glial fibrillary acidic protein
GO: Gene ontology
HDAC1: Histone deacetylase
Iba1: Ionized calcium binding adapter molecule 1
IL-1β: Interleukin 1β
KO: Knockout
LPS: Lipopolysaccharide
NF: Neurofilament-L
NGS: Next-generation sequencing
NMJ: Neuromuscular junction
PBS: Phosphate buffer saline
PFA: Paraformaldehyde
PVDF: Polyvinylidene fluoride
RT-PCR: Reverse transcription-polymerase chain reaction
SA: Sodium arsenite
SDS-PAGE: Sodium dodecyl sulphate-polyacrylamide gel electrophoresis
SG: Stress granule
SOD1: Superoxide dismutase 1
SPF: Specific pathogen free
Syn: Synapsin-1
TA: Tibialis anterior
TDP43: TAR DNA-binding protein 43
TNF-α: Tumor necrosis factor α
TSA: Trichostatin A
WT: Wild-type
YB1: Y-box binding protein 1
