# Supplementary material for: Loss of CREST leads to neuroinflammatory responses and ALS-like motor defects in mice
Source: Transl Neurodegener. 2019 Apr 2;8:13. doi: 10.1186/s40035-019-0152-1 (PMC6444434; doi:10.1186/s40035-019-0152-1)
Supplement: Supplementary file 5 — Table S1. Key Resources Table. (DOCX 74 kb) [file 40035_2019_152_MOESM1_ESM.docx]

**Table S1. Key Resources Table**

| **Reagent or Resource** | **Source** | **Identifier** |
| --- | --- | --- |
| **Antibodies** | | |
| CREST (Rabbit) | Willget | N/A |
| HA | Covance | Cat#MMS-101R, RRID: AB_291262 |
| β-Actin | Abmart | Cat#P30002M |
| BRG1 | Santa Cruz | Cat#sc-17796, RRID: AB_626762 |
| GAPDH | Abcam | Cat#ab8245, RRID: AB_2107448 |
| FUS | Abcam | Cat#ab124923, RRID: AB_10972861 |
| TDP43 | Cell Signaling | Cat#3448S, RRID: AB_2271509 |
| CREST (Goat) | Santa Cruz | Cat#sc-50912, RRID: AB_2195163 |
| Iba1 | Wako | Cat#019-19741, RRID: AB_839504 |
| ChAT | Millipore | Cat#AB144P, RRID: AB_2079751 |
| Neurofilament-L | Cell Signaling | Cat#2837, RRID: AB_823575 |
| Synapsin-1 | Cell Signaling | Cat#D1265 |
| YB1 | Abcam | Cat#ab76149, RRID: AB_2219276 |
| GFP | Invitrogen | Cat#A-11122, RRID: AB_221569 |
| SMI312 | BioLegend | Cat#837904, RRID: AB_2566782 |
| GFAP | Millipore | Cat#AB5541, RRID: AB_177521 |
| HDAC1 | Abcam | Cat#ab7028, RRID: AB_305705 |
| CD68 | Abcam | Cat#ab53444, RRID: AB_869007 |
| Donkey anti-rabbit HRP | GE-Healthcare | Cat#NA934V |
| Sheep anti-mouse HRP | GE-Healthcare | Cat#NA931V |
| Donkey anti-rabbit CF488A conjugate | Biotium | Cat#20015, RRID: AB_10559669 |
| Donkey anti-rat CF488A conjugate | Invitrogen | Cat#A-21208, RRID: AB_141709 |
| Donkey anti-rabbit CF555 conjugate | Biotium | Cat#20038, RRID: AB_10558011 |
| Donkey anti-mouse CF555 conjugate | Biotium | Cat#20037, RRID: AB_10559035 |
| Donkey anti-goat CF555 conjugate | Biotium | Cat#20039, RRID: AB_10556967 |
| Goat anti-chicken CF555 conjugate | Biotium | Cat#20034, RRID: AB_10853135 |
| **Chemicals, Peptides, and Recombinant Proteins** | | |
| Papain | Worthington | Cat#LS003126 |
| Neurobasal medium | Gibco | Cat#21103-049 |
| B27 | Gibco | Cat#17504-044 |
| Protease inhibitor cocktail tablets | Roche | Cat#04693159001 |
| ECL Western Blotting Substrate | Pierce | Cat#32106 |
| Paraformaldehyde (PFA) | Sigma-Aldrich | Cat#P6148 |
| Triton X-100 | Sigma-Aldrich | Cat#T9284 |
| Trizol reagent | Invitrogen | Cat#15596-018 |
| Trichostatin A (TSA) | Cell Signaling | Cat#9950S |
| α-bungarotoxin (BTX) CF488A conjugate | Biotium | Cat#00005 |
| Sodium arsenite (SA) | Sigma-Aldrich | Cat#S7400 |
| **Critical Commercial Assays** | | |
| PrimeScript RT Master Mix | TaKaRa | Cat#RR036A |
| SYBR green premix | Toyobo | Cat#QPK-201 |
| KOD-Plus-Mutagenesis Kit | Toyobo | Cat#SMK-101 |
| ChIP Assay Kit | Upstate | Cat#17-295 |
| **Experimental Models: Organisms/Strains** | | |
| Mouse: C57BL/6 | JAX | RRID: IMSR_JAX: 000664 |
| **Oligonucleotides** | | |
| Forward primer for CREST Q388X cDNA mutagenesis:  5’-TAGGGCCAGTATGGAAATTACCAGCA-3’ | This paper | N/A |
| Reverse primer for CREST Q388X cDNA mutagenesis:  5’-TTCATAGCCGTAGGGCCGCTG-3’ | This paper | N/A |
| shRNA targeting sequence of mouse CREST:  5’-GGTCAGCAGTATGGAAGCT-3’ | Qiu and Ghosh, 2008 | N/A |
| shRNA targeting sequence of mouse BRG1:  5’-CCAAAGCAACCATCGAACT-3’ | Qiu and Ghosh, 2008 | N/A |
| CRISPR/Cas9 sgRNA sequence for KO mice:  5’-ATGCAGGGCCAGATCGGTAA-3’ | This paper | N/A |
| CRISPR/Cas9 sgRNA sequence for Q394X mice:  5’-GCGGCCTTACGGCTATGAAC-3’ | This paper | N/A |
| Repair donor for Q394X mice construction with CRISPR/Cas9:  5’-CATCGCAGACGGGACCTTCTGCCCAGCAGCAGCGGCCTTACGGCTATGAATGAGCAAGCTTTCTGGGCGTTTCAGGAAGCGCTATCTGCCAAGTGTCAAGTGA-3’ | This paper | N/A |
| Primer for qPCR: *Il1b* Forward:  5’-TGCCACCTTTTGACAGTGATG-3’ | This paper | N/A |
| Primer for qPCR: *Il1b* Reverse:  5’-TGATGTGCTGCTGCGAGATT-3’ | This paper | N/A |
| Primer for qPCR: *Tnfa* Forward:  5’-ACTTCGGGGTGATCGGTCCCC-3’ | This paper | N/A |
| Primer for qPCR: *Tnfa* Reverse:  5’-TGGTTTGCTACGACGTGGGCTAC-3’ | This paper | N/A |
| Primer for qPCR: *Cox-2* Forward:  5’-CCCTGCTGCCCGACACCTTC-3’ | This paper | N/A |
| Primer for qPCR: *Cox-2* Reverse:  5’-CCAGCAACCCGGCCAGCAAT-3’ | This paper | N/A |
| Primer for qPCR: *Cxcl10* Forward:  5’-AAGTGCTGCCGTCATTTTCT-3’ | This paper | N/A |
| Primer for qPCR: *Cxcl10* Reverse:  5’-CCTATGGCCCTCATTCTCAC-3’ | This paper | N/A |
| Primer for qPCR: *Ccl2* Forward:  5’-TTAAAAACCTGGATCGGAACCAA-3’ | This paper | N/A |
| Primer for qPCR: *Ccl2* Reverse:  5’-GCATTAGCTTCAGATTTACGGGT-3’ | This paper | N/A |
| Primer for qPCR: *Actb* Forward:  5’-GGCTCCTAGCACCATGAAGAT-3’ | This paper | N/A |
| Primer for qPCR: *Actb* Reverse:  5’-TAAAACGCAGCTCAGTAACAGT-3’ | This paper | N/A |
| QPCR Primer for ChIP: *Cxcl10* Forward:  5’-GCAATGCCCTCGGTTTACAG-3’ | This paper | N/A |
| QPCR Primer for ChIP: *Cxcl10* Reverse:  5’-TCTGCAAAGAGTTTCCCTCCC-3’ | This paper | N/A |
| QPCR Primer for ChIP: *Ccl2* Forward:  5’-CACTTCCTGGAAACACCCGA-3’ | This paper | N/A |
| QPCR Primer for ChIP: *Ccl2* Reverse:  5’-CTGCTCTGAGGCAGCCTTTT-3’ | This paper | N/A |
| QPCR Primer for ChIP: *c-Fos* Forward:  5’-GAAAGCCTGGGGCGTAGAG-3’ | This paper | N/A |
| QPCR Primer for ChIP: *c-Fos* Reverse:  5’-CCTCAGCTGGCGCCTTTAT-3’ | This paper | N/A |
| **Software and Algorithms** | | |
| ImageJ | NIH | RRID: SCR_003070 |
| GraphPad Prism 6 | GraphPad Software | RRID: SCR_002798 |
| EthoVision XT | Noldus | RRID: SCR_000441 |
| CatWalk XT | Noldus | N/A |
| StepOnePlus Real-Time PCR System | Applied Biosystems | RRID: SCR_015805 |
| DAVID Bioinformatics Resources | Leidos Biomedical Research, Inc. | RRID: SCR_001881 |
| **Databases** | | |
| Brain RNA-Seq | Zhang et al., 2014 | RRID: SCR_013736 |
