# Supplementary figures and images for: Loss of CREST leads to neuroinflammatory responses and ALS-like motor defects in mice
Source: Transl Neurodegener. 2019 Apr 2;8:13. doi: 10.1186/s40035-019-0152-1 (PMC6444434; doi:10.1186/s40035-019-0152-1)

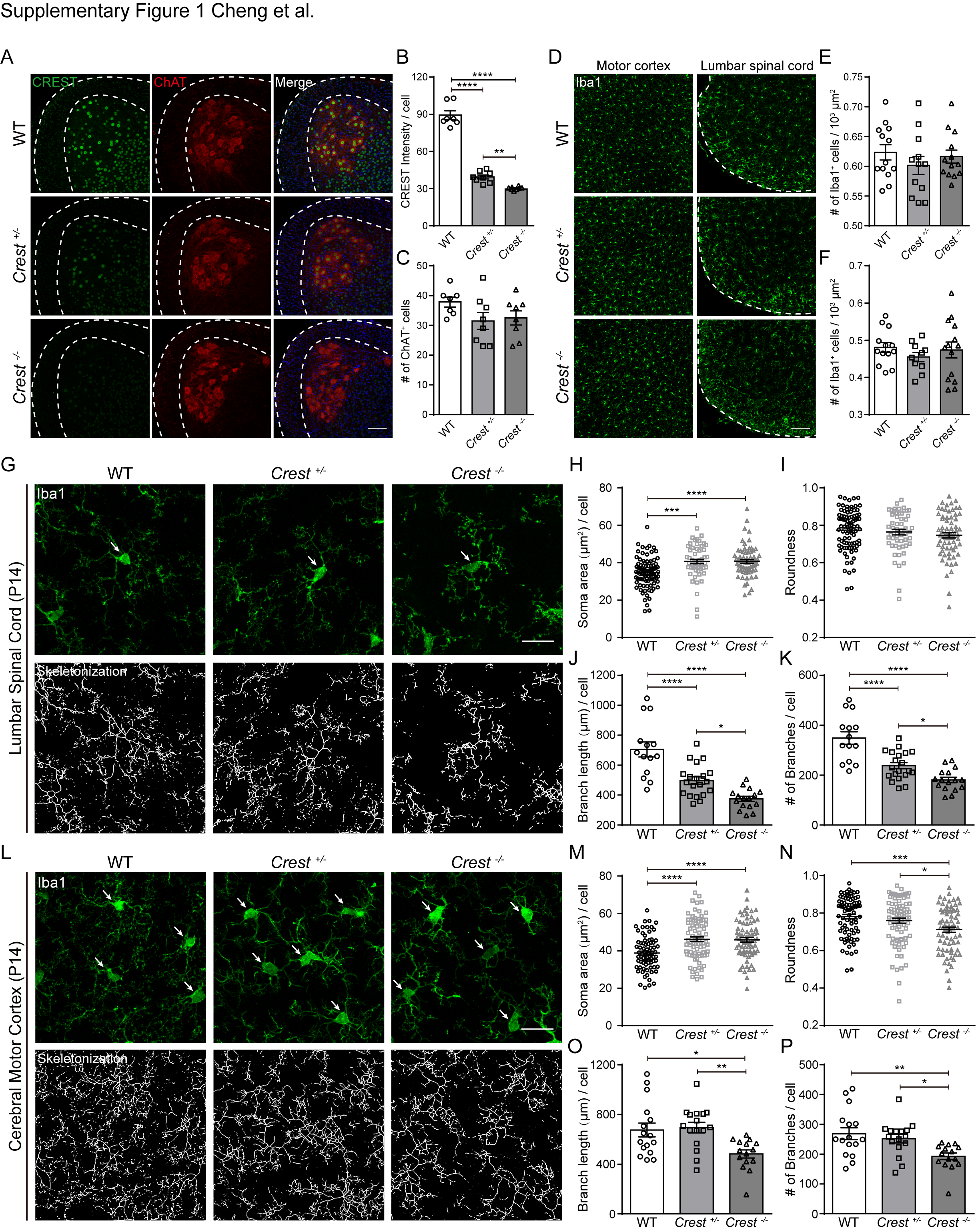

Supplement: Supplementary file 6 — CREST KO Mice Show the Activation of Microglia in CNS at P14, related to Fig. 2. (A) Representative confocal immunohistochemistry images of CREST-positive cells (green) and ChAT-positive motor neurons (red) in the lumbar spinal cords. Dashed lines divide the white and grey matter of lumbar spinal cord. Scale bar, 100μm. (B and C) Quantification of CREST intensities (B) and the number of ChAT-positive motor neurons (C) in the lumbar spinal cords. Each symbol indicates one image. (D) Representative confocal immunohistochemistry images of Iba1-positive microglia (green) in cerebral motor cortices (left panels) and in lumbar spinal cords (right panels). Dashed lines show the lumbar spinal cords. Scale bar, 100μm. (E and F) Quantification of Iba1-positive microglia (green) in cerebral motor cortices (E) and in lumbar spinal cords (F). Each symbol indicates one image. (G) Representative immunohistochemistry images of Iba1-positive (green; arrows) microglia (top panels) and their skeletonized appearance (bottom panels) in the lumbar spinal cords. Scale bar, 20μm. (H-K) Quantification of microglial morphological parameters including soma area (H) and roundness (I) of the projection of Iba1-positive cell bodies (each symbol indicating one cell), and branch length (J) and branch number (K) per cell (each symbol indicating one image, about 6 images per mouse) in the lumbar spinal cords. (L) Representative immunohistochemistry images of Iba1-positive (green; arrows) microglia (top panels) and their skeletonized appearance (bottom panels) in the cerebral motor cortices. Scale bar, 20μm. (M-P) Quantification of microglial morphological parameters including soma area (M) and roundness (N) of the projection of Iba1-positive cell bodies (each symbol indicating one cell), and branch length (O) and branch number (P) per cell (each symbol indicating one image, about 6 images per mouse) in the cerebral motor cortices. Error bars represent SEM. *p < 0.05, **p < 0.01, ***p < 0.001, and ** [file 40035_2019_152_MOESM2_ESM.tif]

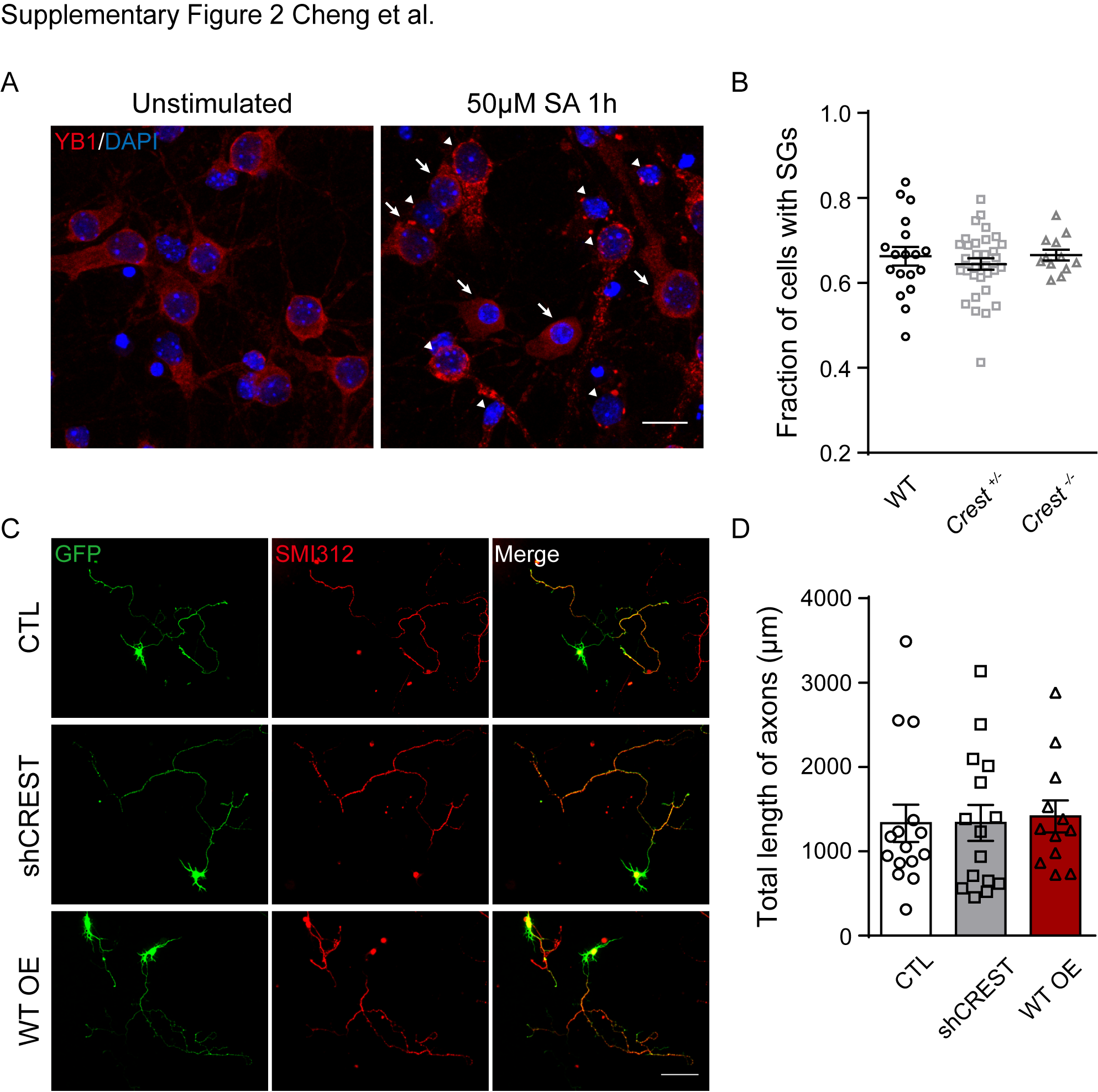

Supplement: Supplementary file 7 — Figure S2. Loss of CREST Function Does not Affect the Formation of Stress Granules and the Axon Length in Cultured Neurons in vitro, related to Fig. 2. (A) Representative Immunofluorescence staining of stress granule marker YB1 (red) in the treatment of nothing as control (left) or 50μM sodium arsenite (SA) for 1h (right). Arrows indicate the cells without stress granules (SGs) in the treatment of SA. Arrowheads indicate the cells with SGs. Scale bar, 15μm. (B) Quantification of the fraction of cells with SGs in cultured neurons isolated from embryotic Crest −/−, Crest +/− mice and WT littermates. (C and D) Representative Immunofluorescence images (C) and the total length quantification (D) of SMI312-positive (red) axons in cultured neurons expressing GFP (C, top), shCREST labeled by GFP (C, middle) and WT human CREST labeled by GFP (green) (C, bottom). Scale bar, 100μm. Error bars represent SEM. One-way ANOVA. (TIF 13203 kb) [file 40035_2019_152_MOESM3_ESM.tif]

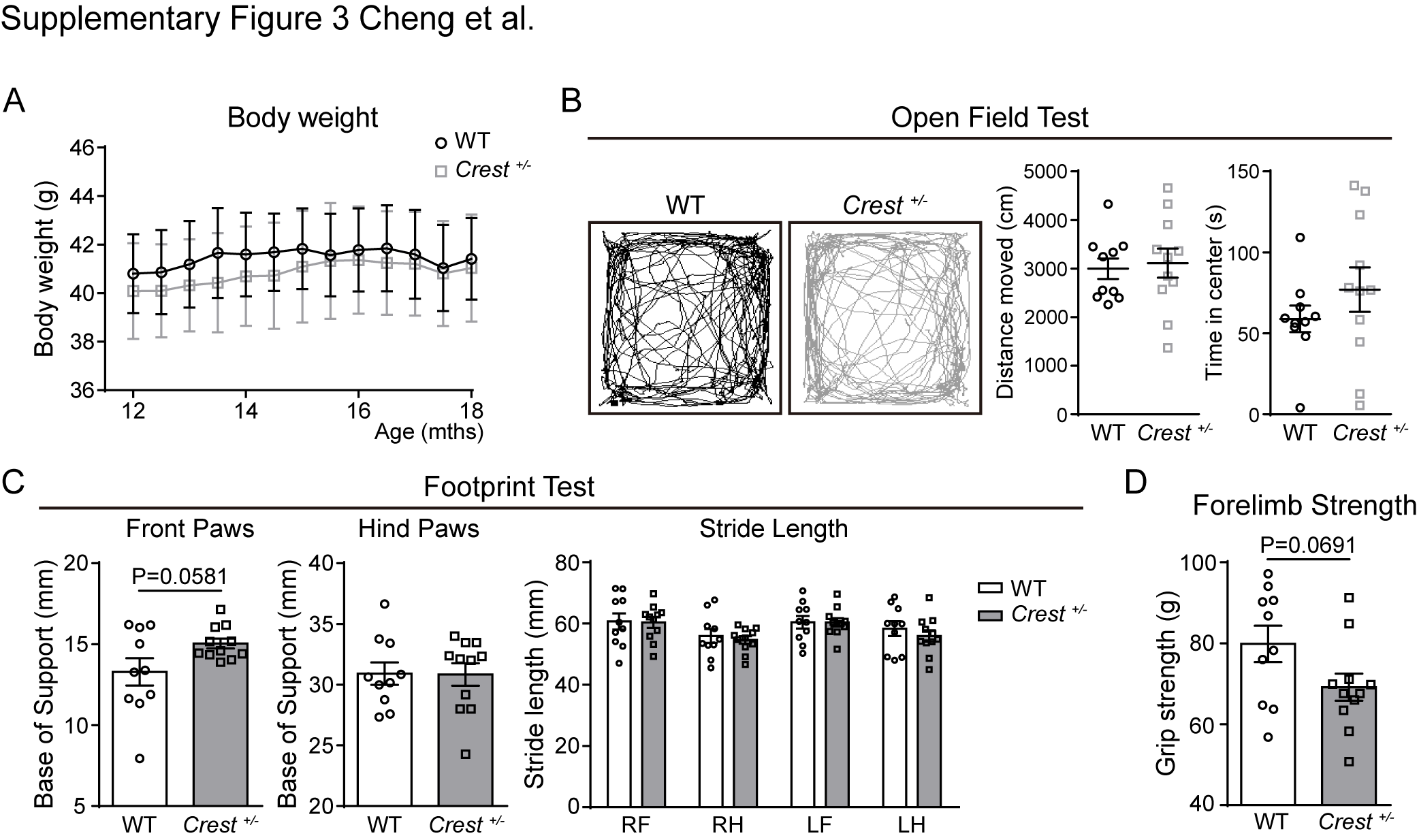

Supplement: Supplementary file 8 — Figure S3. Behavioral Tests of Motor Phenotypes on Crest +/− Mice, related to Fig. 4. (A) Body weight of Crest +/− mice (n = 11) and WT littermates (n = 9) from the age of 12 months to 18 months. The measurement was performed every 2 weeks. (B) Representative traces (two left panels), the moving distance (middle graph) and the center staying time (right graph) of Crest +/− mice (n = 11) and WT littermates (n = 9) at 18 months in open field tests. The duration of each trial was 10 min. (C) Footprint tests showing the width of support bases of both front paws (left graph) and hind paws (middle graph), and the length of strides of four paws (right graph) of Crest +/− mice (n = 11) and WT littermates (n = 9) at 18 months. (D) Grip strength tests showing the forelimb strength of Crest +/− mice (n = 11) and WT littermates (n = 9) at 18 months. Error bars represent SEM. Student’s t test. (TIF 8574 kb) [file 40035_2019_152_MOESM4_ESM.tif]

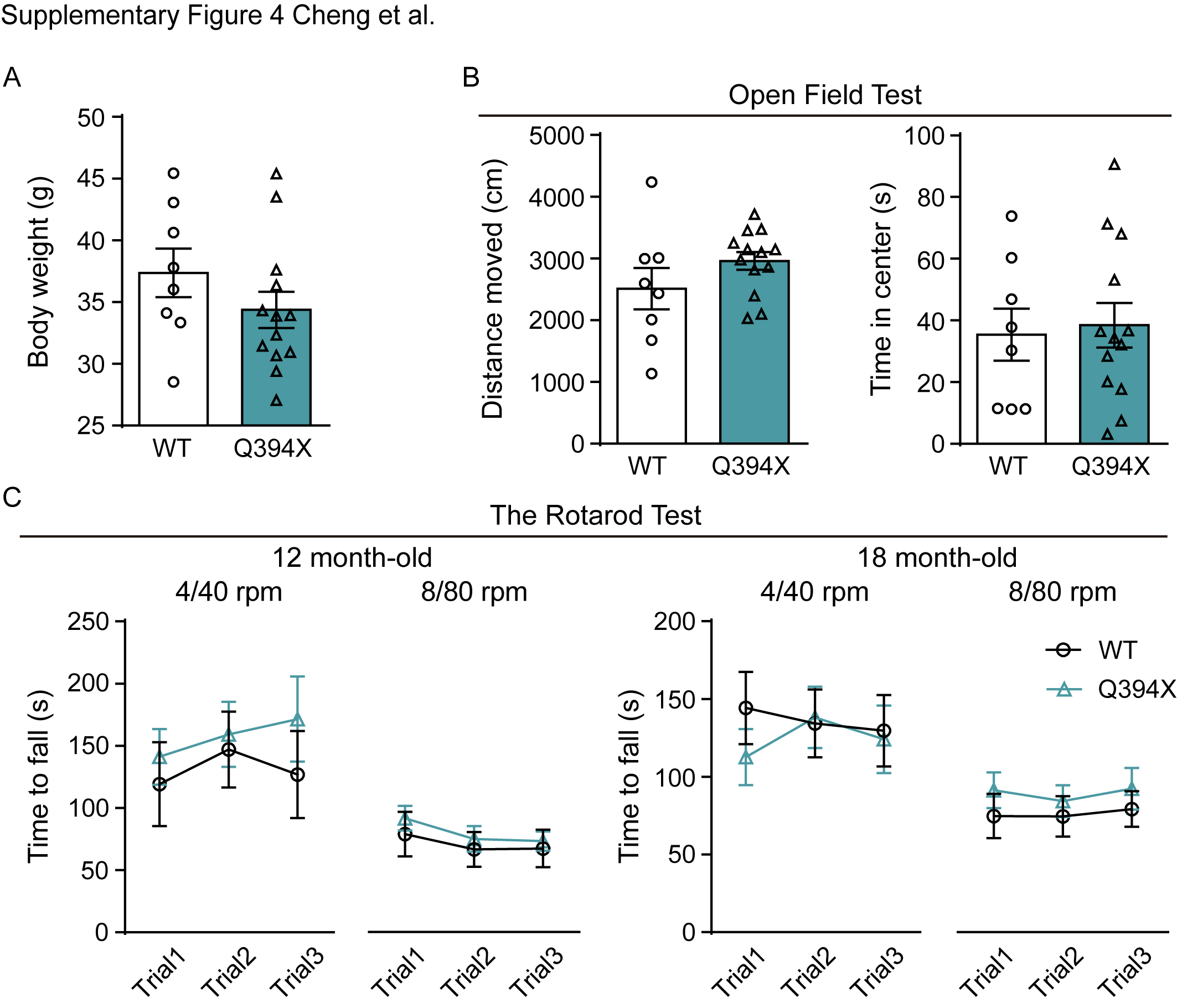

Supplement: Supplementary file 9 — Figure S4. Behavioral Tests of Motor Phenotypes on Q394X Mice, related to Fig. 4. (A) Body weight of Q394X mice (n = 13) and WT littermates (n = 8) at 18 months. (B) Open field tests showing the moving distance (left graph) and the center staying time (right graph) of Q394X mice (n = 13) and WT littermates (n = 8) at 18 months. The duration of each trial was 10 min. (C) Rotarod tests performed with two accelerating modes (4 to 40 rpm and 8 to 80 rpm in 300 s) for 3 trials on Q394X mice (n = 13) and WT littermates (n = 8) at the age of 12 months (two left graphs) and 18 months (two right graphs). Error bars represent SEM. Student’s t test. (TIF 12255 kb) [file 40035_2019_152_MOESM5_ESM.tif]

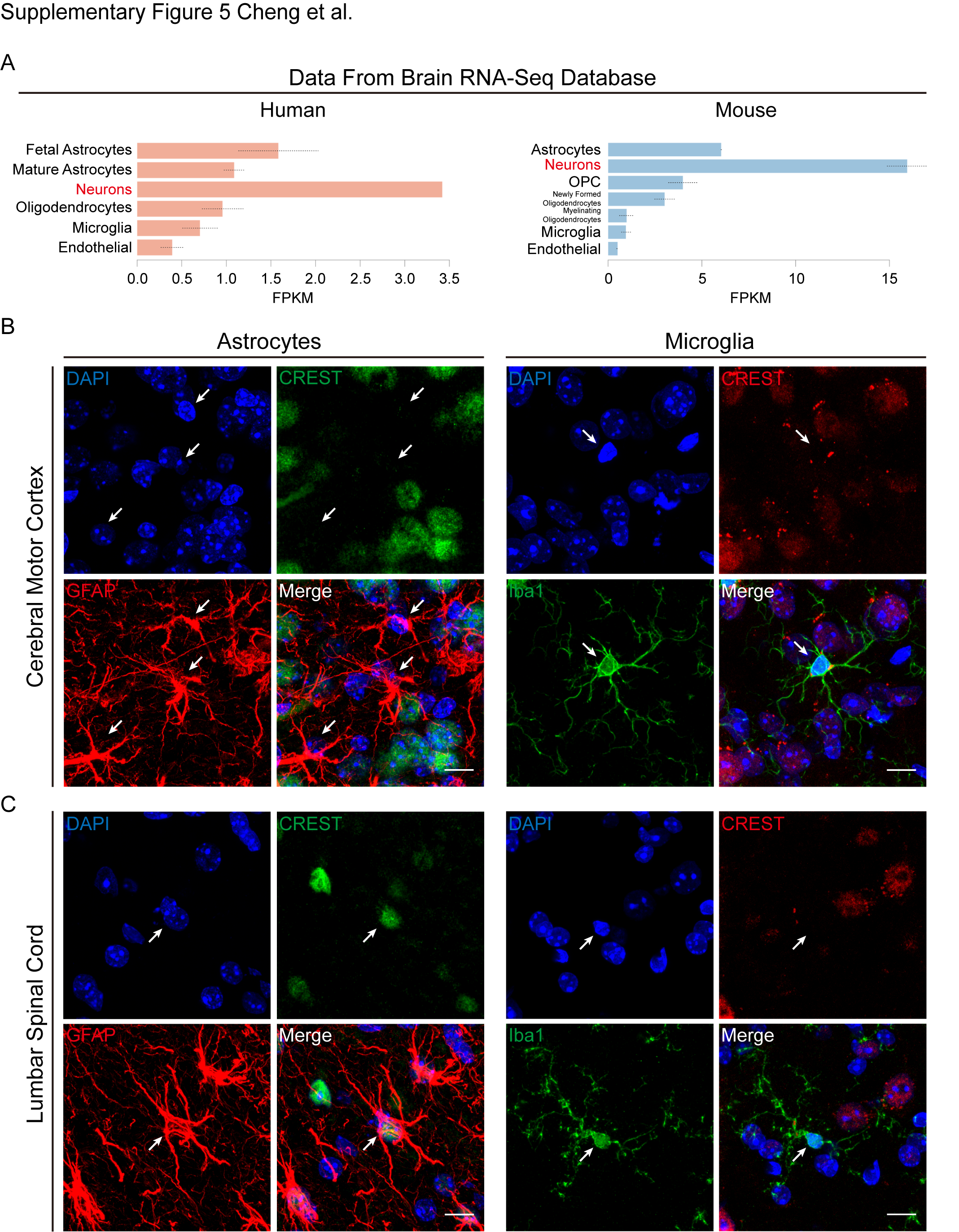

Supplement: Supplementary file 10 — Figure S5. Expression Pattern of CREST in CNS, related to Fig. 5. (A) Relative expression of CREST in cell types of human (left) and mouse (right) brain revealed by Brain RNA-Seq database. (B and C) Representative confocal immunohistochemistry images of co-localization of astrocyte marker GFAP (red) and CREST (green) (four left panels), and of microglial marker Iba1 (green) and CREST (red) (four right panels) in both cerebral motor cortex (B) and lumbar spinal cord (C). Arrows indicate GFAP-positive astrocytes or Iba1-positive microglia. Scale bar, 10μm. (TIF 21670 kb) [file 40035_2019_152_MOESM6_ESM.tif]

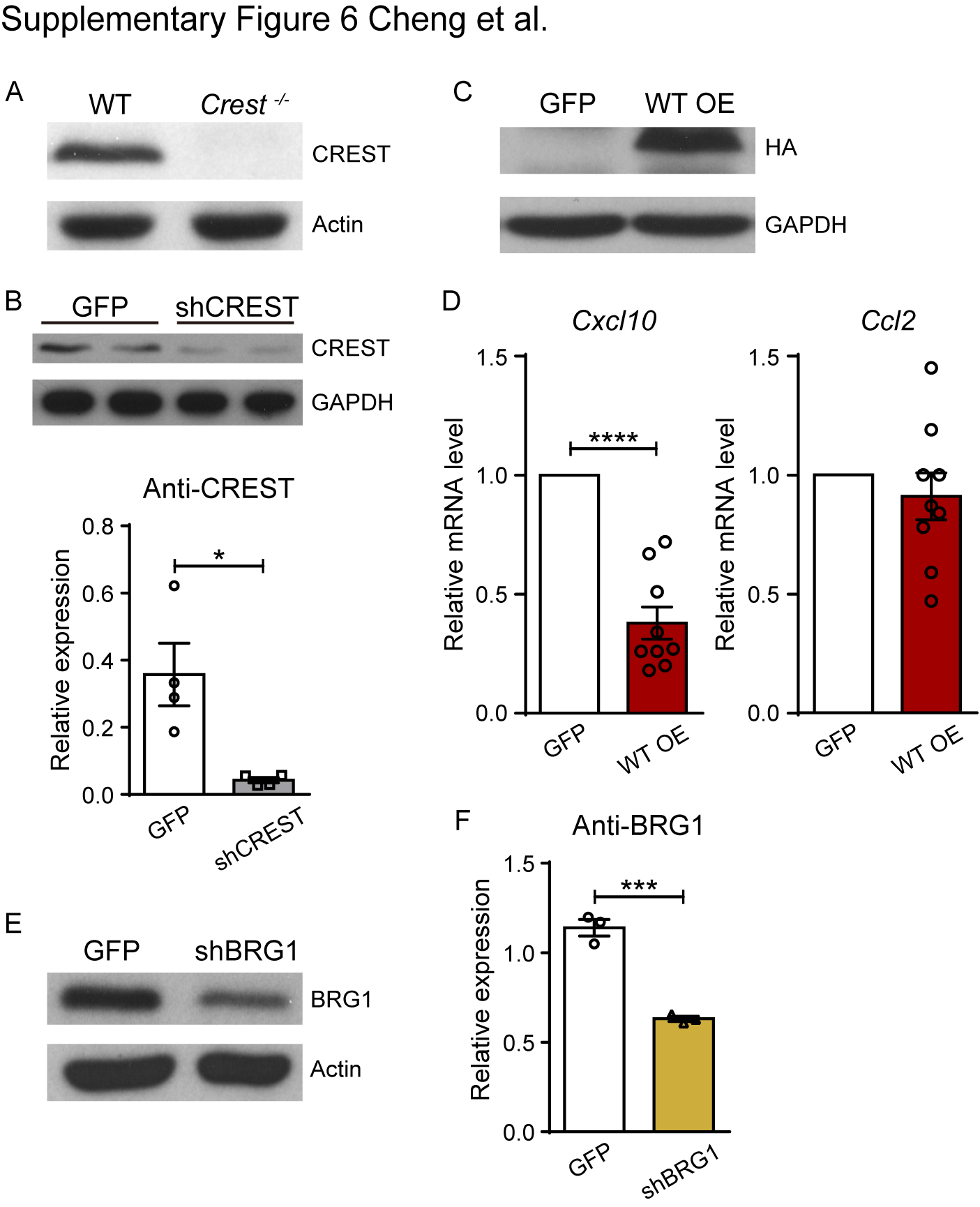

Supplement: Supplementary file 11 — Figure S6. Validation of Knockdown Efficiency of shRNAs and Inhibitory Effect of CREST on Transcription of Cxcl10 in Cultured Neurons, related to Fig. 5. (A) Immunoblot of protein samples from primary cortical neurons isolated from embryotic Crest −/− and WT littermates, using anti-CREST (top) and anti-Actin (bottom) antibodies. (B) Immunoblot and intensity quantification of protein from primary cortical neurons infected with lentivirus carrying CREST shRNA (shCREST) and GFP (as control). Relative expression represents the intensity ratios of CREST/GAPDH. (C) Immunoblot of protein from primary cortical neurons infected with lentivirus expressing GFP or HA-tagged WT CREST, using anti-HA (top) and anti-GAPDH (bottom) antibodies. (D) Quantitative RT-PCR of Cxcl10 (left) and Ccl2 (right) in primary cortical neurons infected with lentivirus expressing GFP or HA-tagged WT CREST. (E and F) Immunoblot (E) and quantification (F) of protein from primary cortical neurons infected with lentivirus carrying BRG1 shRNA (shBRG1) and GFP (as control). Relative expression represents the intensity ratios of BRG1/Actin normalized to one control sample. Error bars represent SEM. *p < 0.05, ***p < 0.001, and ****p < 0.0001, Student’s t test. (TIF 8284 kb) [file 40035_2019_152_MOESM7_ESM.tif]
